# Site‐Specific Carboxylate Functionalization in Conjugated Hole Transport Polymers Enables Efficient CsPbI_2_Br Single‐Junction and Tandem Solar Cells

**DOI:** 10.1002/advs.202524013

**Published:** 2026-02-15

**Authors:** Jingfei Wang, Weilin Zhang, Haobin Zhang, Zongwei Chen, Tangyue Xue, Qing Guo, Zhi Zheng, Zongtao Wang, Erjun Zhou, Qiang Guo

**Affiliations:** ^1^ Henan Institute of Advanced Technology Zhengzhou University Zhengzhou China; ^2^ College of Biological and Chemical Engineering Jiaxing University Jiaxing China; ^3^ College of Chemical and Materials Engineering Xuchang University Xuchang Henan China

**Keywords:** conjugated polymers, CsPbI_2_Br, hole transport materials, isomeric sites, perovskite solar cells

## Abstract

Hole transport materials (HTMs) are crucial for achieving high‐efficiency and stable all‐inorganic CsPbI_2_Br perovskite solar cells (PSCs). While carboxylate‐functionalized polymers have shown considerable promise, the influence of different carboxylate substitution positions on device performance remains poorly understood. In this work, we designed and synthesized two polymeric isomers (2TC‐F and TTC‐F) to systematically investigated the effects of connecting the carboxylate side chain at different sites of the thiophene (T) and thieno[3,4‐b]thiophene (TT) units on the energy level structure, molecular packing behavior, hole mobility, and charge transfer properties at the perovskite/HTM interface. The study demonstrates that, compared to 2TC‐F, TTC‐F exhibits better energy level alignment, higher planarity, and more effective defect passivation. As a result, TTC‐F‐based CsPbI_2_Br PSCs achieved a power conversion efficiency (PCE) of 17.58%, significantly outperforming the 2TC‐F‐based device (14.54%), along with excellent stability under thermal aging and ambient storage. When integrated into perovskite/organic tandem solar cells (TSCs), the TTC‐F‐based device reached a PCE of 23.29%. This work highlights the importance of site‐specific side‐chain engineering in designing high‐performance HTMs.

## Graphical abstract

Two polymeric isomers, 2TC‐F and TTC‐F, are designed and synthesized as hole transport materials to investigate the impact of the carboxylate side‐chain connection site. The TTC‐F isomer, owing to its superior energy level alignment, higher planarity, and more effective defect passivation, enabled CsPbI_2_Br single‐junction and CsPbI_2_Br/D18:Y6 tandem solar cells to achieve high power conversion efficiencies of 17.58% and 23.29%, respectively.

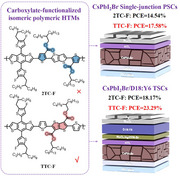

## 1 Introduction

Organic‐inorganic halide perovskite solar cells (PSCs) have garnered significant attention in the photovoltaic community due to their remarkably improved power conversion efficiencies (PCEs). However, its operational stability remains a major challenge for commercialization. In this context, all‐inorganic perovskites such as CsPbX_3_ (X═I, Br) have attracted considerable attention owing to their excellent thermal and photostability, offering a potential pathway to addressing stability concerns [1, 2, 3, 4, 5]. Among them, the mixed‐halide composition CsPbI_2_Br achieves an optimal balance: it maintains a desirable optical bandgap of around 1.91 eV, while overcoming the poor phase stability of CsPbI_3_ and the overly wide bandgap of CsPbBr_3_ [6, 7, 8]. These attributes result in enhanced environmental stability, making it particularly suitable for application as a wide‐bandgap top cell in tandem devices. In previously reported studies, CsPbI_2_Br has been widely utilized in perovskite/organic tandem solar cells (TSCs) [9, 10]. Compared to other TSC architectures, perovskite/organic TSCs exhibit a unique advantage: the active layer of the organic sub‐cell is typically deposited from non‐polar solvents (e.g., chlorobenzene, chloroform), which are orthogonal to those used for perovskite film fabrication. This solvent orthogonality significantly relaxes the constraints on the selection of the interconnection layer (ICL), thereby enhancing the flexibility of tandem structure design [11]. Furthermore, the strong near‐infrared absorption of low‐bandgap organic materials enables the rear sub‐cell to achieve high performance even at a minimal thickness of approximately 100 nm. This feature notably enhances the potential of such tandem configurations for applications in flexible photovoltaics [12].

The hole transport layer (HTL) is critical for PSC performance, responsible for efficient hole extraction, interfacial recombination suppression, and perovskite protection [13, 14, 15]. Organic hole transport materials (HTMs) are primarily categorized into small molecules and polymers. Among small molecule HTMs, Spiro‐OMeTAD is the most commonly utilized, but it usually relies on highly hygroscopic and easily mobile chemical dopants to enhance conductivity, which poses a significant challenge to the long‐term stability of the devices [16, 17]. In contrast, polymeric HTMs have attracted increasing attention due to their excellent film‐forming properties, good mechanical flexibility, and potential for dopant‐free applications. In the molecular design of polymeric HTMs, donor‐acceptor (D‐A) and pure‐donor (D) types represent two mainstream structural strategies. D‐A type polymers can effectively modulate energy levels and enhance charge carrier mobility through the intramolecular charge transfer (ICT) effect, and various D‐A polymers have demonstrated promising performance in PSCs [18, 19, 20, 21, 22]. However, the introduction of strongly electron‐withdrawing A‐units may lead to an excessively low lowest unoccupied molecular orbital (LUMO) level, weakening the material's electron‐blocking capability and thereby increasing the probability of interfacial recombination. Moreover, the complex synthesis of A‐units and their intrinsic instability also limit further development and application of such materials. In comparison, pure‐D type polymers generally exhibit higher highest occupied molecular orbital (HOMO) and LUMO levels, which not only facilitate efficient hole extraction but also effectively block electron transport toward the electrode, serving a dual interfacial role. Additionally, the absence of less stable A‐units in their structure results in superior intrinsic stability [23, 24].

Thiophene and benzene rings, as classic donor units for constructing high‐mobility conjugated polymers, have been widely employed in pure‐D type HTMs such as P3HT and PTAA, which exhibit excellent performance in PSCs [25, 26, 27]. Nevertheless, when applied in CsPbI_2_Br PSCs, the relatively high HOMO levels of P3HT and PTAA, while beneficial for hole extraction, lead to considerable open‐circuit voltage (*V*_OC_) loss. Thus, the development of pure‐D polymeric HTMs with deeper HOMO levels is of great research significance for CsPbI_2_Br PSCs. Side‐chain functionalization is a key strategy for optimizing polymers’ properties. Without altering the backbone conjugation, introducing specific functional groups into the side chains enables fine‐tuning of energy levels, enhancement of molecular planarity and crystallinity, and effective passivation of interfacial defects [18, 28, 29]. Among various functional groups, the carboxylate group has shown tremendous potential in side‐chain engineering, owing to its moderate electron‐withdrawing nature and the ability of its carbonyl (C═O) group to coordinate with undercoordinated Pb^2+^ ions in the perovskite [30, 31]. For instance, Zhang et al. reported a straightforward and readily synthesizable polythiophene derivative, PDCBT. By introducing an electron‐withdrawing carboxylate group into the side chain, its HOMO level was lowered from −4.90 eV for P3HT to −5.26 eV [32]. The lower HOMO level of PDCBT allows for better energy level matching with CsPbI_2_Br. Moreover, the carboxylate group can coordinate and passivate undercoordinated Pb^2^^+^ ion defects in perovskites, allowing CsPbI_2_Br PSCs based on PDCBT HTM to achieve high *V*_OC_ [33]. Furthermore, other polymer HTMs featuring carboxylate‐functionalized side chains, such as PTB7‐Th [34], PTVT‐T [35], and TTC‐Cl [36], have also exhibited excellent performance in PSCs, further validating the effectiveness of the carboxylate‐group functionalization strategy. Although previous studies have preliminarily revealed the positive effects of carboxylate groups, research on how their precise positioning in polymer side chains systematically affects the photovoltaic performance of devices remains relatively limited.

**FIGURE 1:**
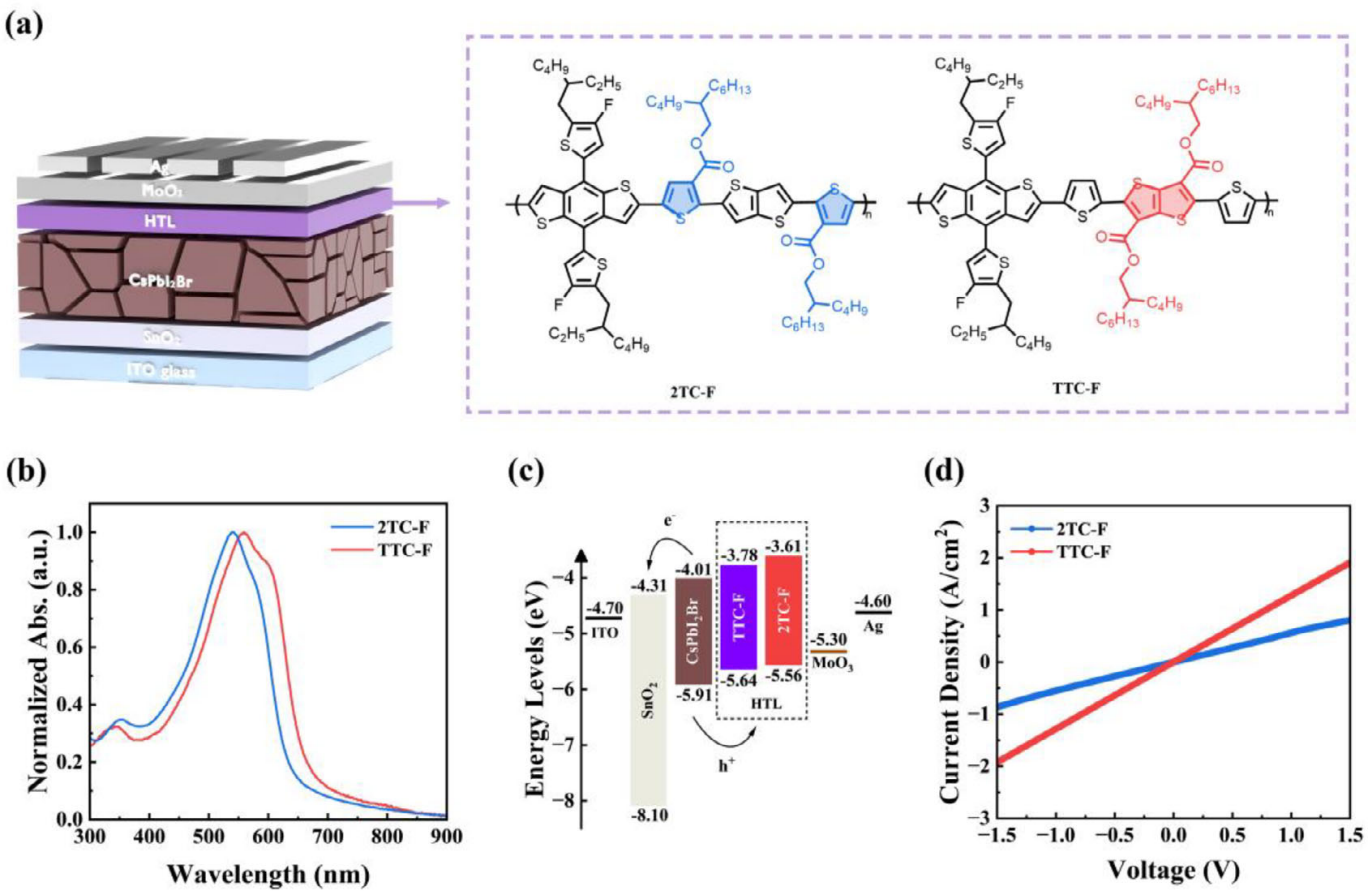
(a) Device structure of the CsPbI_2_Br PSCs and the molecular structures of 2TC‐F and TTC‐F; (b) UV–vis absorption spectra of 2TC‐F and TTC‐F films; (c) Energy level diagram of the CsPbI_2_Br PSCs; (d) *J–V* curves of the ITO/HTM/Ag devices.

In order to investigate the effect of the attachment position of carboxylate side chains on polymer properties and device performance, two pure D‐type conjugated polymers with isomeric carboxylate‐functionalized side chains, namely 2TC‐F and TTC‐F, were designed and synthesized in this work. Both polymers share an identical electron‐donating backbone (benzodithiophene‐thiophene‐thieno[3,4‐b]thiophene), with the key distinction lying in the positioning of the carboxylate side chain: it is attached to the T unit in 2TC‐F and to the TT unit in TTC‐F (Figure 1a). The effects of carboxylate substitution positions on the optoelectronic properties, molecular packing, film morphology, defect passivation ability, and charge transport dynamics of polymers were studied. Results show that CsPbI_2_Br PSCs based on TTC‐F HTM achieved a PCE of 17.58%, significantly surpassing that of devices based on 2TC‐F HTM (14.54%). Moreover, TTC‐F‐based devices demonstrated excellent stability. In addition, we fabricated perovskite (CsPbI_2_Br)/organic (D18:Y6) TSCs using TTC‐F/MoO_3_/Ag/PFN‐Br and 2TC‐F/MoO_3_/Ag/PFN‐Br as ICLs, achieving PCEs of 23.29% and 18.17%, respectively. These results confirm that TTC‐F outperforms 2TC‐F not only as an HTM but also as a component in ICLs.

## 2 Results and Discussion

To investigate the backbone conformational characteristics, we performed geometric structure optimizations on the repeat units of the two polymers using density functional theory (DFT). For computational simplicity, all alkyl chains have been replaced with methyl groups, and the bonding sites of the repeating units have been saturated with hydrogen atoms to simulate the electronic structure in an infinitely long chain. From the top view and side view (Figure S1), it can be observed that in 2TC‐F, significant steric hindrance exists between the sulfur atom in the thiophene ring and the oxygen atom in the carboxylate group. This leads to a distortion of the molecular backbone, which is unfavorable for achieving ordered molecular stacking. In contrast, the main chain of TTC‐F exhibits an excellent linear conformation and planarity, facilitating the formation of a closely packed and orderly solid‐state stacking structure [37]. The normalized UV–vis absorption spectra are shown in Figure 1b, with optical parameters summarized in Table S1. Both polymers exhibit weak absorption bands in the 300–400 nm wavelength range, attributed to the *π–π* ^*^ transitions of the polymer main chain; whereas in the 450–700 nm range, broad and relatively strong absorption bands appear, corresponding to the ICT process [38]. Notably, the film‐state absorption of TTC‐F is redshifted compared to that of 2TC‐F in the 450–650 nm region. This redshift suggests that the altered carboxylate position in TTC‐F optimizes molecular conformation, enhances backbone planarity, and strengthens interchain *π–π* stacking interactions [39, 40, 41, 42]. Subsequently, the electrochemical behavior of the polymers was characterized in an acetonitrile solution using cyclic voltammetry (CV), with Fc/Fc^+^ employed as the reference electrode. Figure S2 presents the CV test curves, and the corresponding calculated results are summarized in Table S1. The initial oxidation potentials (φ_ox_) of 2TC‐F and TTC‐F were measured as 1.31 and 1.39 V, respectively. According to $E_{\mathrm{HOMO}}=-e(\varphi_{\mathrm{ox}}-\varphi_{(\mathrm{Fc}/Fc^{+})}+4.8)$, the HOMO energy levels of 2TC‐F and TTC‐F were determined to be −5.56 and −5.64 eV, respectively. Combined with the optical bandgaps ($E_{g}^{\mathrm{opt}}$) from UV–vis spectra, LUMO levels were calculated to be −3.61 and −3.78 eV, respectively. The energy level alignment diagram of the CsPbI_2_Br PSCs with TTC‐F/2TC‐F HTL is illustrated in Figure 1c, where the energy levels of CsPbI_2_Br are taken from literature [19]. The smaller offset between the HOMO of TTC‐F and the valence band maximum of CsPbI_2_Br facilitates efficient hole extraction and transport while minimizing voltage loss at the interface. Hole mobility (μ_h_) and conductivity (σ) were evaluated using the space‐charge limited current (SCLC) method. The hole mobility (μ_h_) value of TTC‐F was determined to be 9.27 × 10^−4^ cm^2^ V^−1^ s^−1^ (Figure S3), higher than that of 2TC‐F (6.72 × 10^−4^ cm^2^ V^−1^ s^−1^). Conductivity tests were performed on ITO/HTL/Ag structures (Figure 1d), revealing a σ value of 6.36 × 10^−6^ S cm^−1^ for TTC‐F, more than twice that of 2TC‐F (2.74 × 10^−6^ S cm^−1^).

**FIGURE 2:**
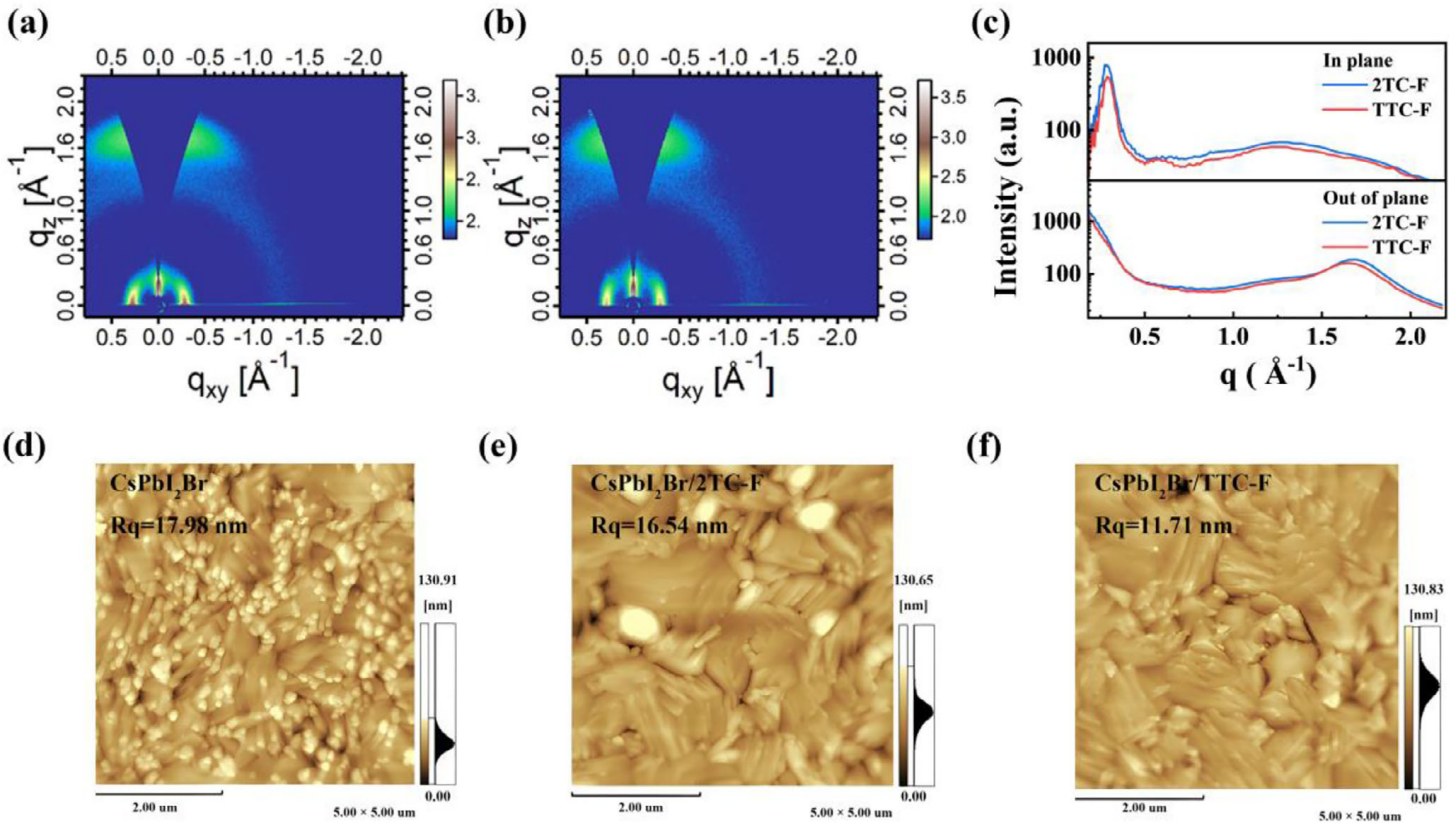
(a) 2D‐GIWAXS pattern of 2TC‐F film; (b) 2D‐GIWAXS pattern of TTC‐F film; (c) Corresponding out‐of‐plane (OOP) and in‐plane (IP) GIWAXS line‐cut profiles; AFM surface topographic images of (d) CsPbI_2_Br, (e) CsPbI_2_Br/2TC‐F, and (f) CsPbI_2_Br/TTC‐F.

Molecular stacking orientation is a critical factor governing charge transport properties in organic semiconductors [43]. The grazing‐incidence wide‐angle X‐ray scattering (GIWAXS) was performed to probe the molecular arrangement and ordering in the HTLs. The corresponding diffraction peak data are summarized in Tables S2 and S3. As shown in Figure 2a–c, both TTC‐F and 2TC‐F exhibit a typical face‐on molecular orientation. Their out‐of‐plane (OOP) diffraction peaks are located at q_z_ = 1.64 Å^−1^ and 1.67 Å^−1^, respectively. According to Bragg's law, the *π–π* stacking distances of TTC‐F and 2TC‐F are 3.83 Å and 3.77 Å, respectively, indicating similar intermolecular spacing in this direction. In the in‐plane (IP) direction, TTC‐F exhibits distinct (100) and (200) diffraction peaks at q_xy_ = 0.29 and 0.58 Å^−1^, respectively, while the higher‐order (200) diffraction peak for 2TC‐F is not pronounced. The IP (100) peak corresponds to a lamellar spacing of 21.59 Å for TTC‐F, smaller than that of 2TC‐F (21.89 Å). Moreover, based on the Scherrer formula, the crystal coherence length (CCL) of TTC‐F is 147.93 Å, larger than that of 2TC‐F (135.26 Å). These results indicate that tuning the carboxylate side‐chain position in TTC‐F promotes more ordered molecular packing and enhanced crystallinity [44, 45], which is conducive to improving hole mobility.

Atomic force microscopy (AFM) was further utilized to characterize the surface morphology and roughness of the CsPbI_2_Br film before and after HTL deposition (Figure 2d–f). The pristine CsPbI_2_Br film exhibits clear light and dark contrast on its surface with considerable undulation, indicating a high roughness. Benefiting from the excellent film‐forming properties of the polymer, the root mean square roughness (Rq) decreased from 17.98 nm for pristine CsPbI_2_Br to 16.54 nm for CsPbI_2_Br/2TC‐F and further to 11.71 nm for CsPbI_2_Br/TTC‐F. In addition to analyzing film morphology, we further investigated the effects of TTC‐F and 2TC‐F on the surface potential distribution through Kelvin Probe Force Microscopy (KPFM) measurements. Figure S4 illustrates a comparison of the potential distribution (CPD) for CsPbI_2_Br, CsPbI_2_Br/2TC‐F, and CsPbI_2_Br/TTC‐F films. The CsPbI_2_Br/TTC‐F films exhibit not only a more uniform surface potential but also a more consistent charge distribution, suggesting a lower surface defect density.

**FIGURE 3:**
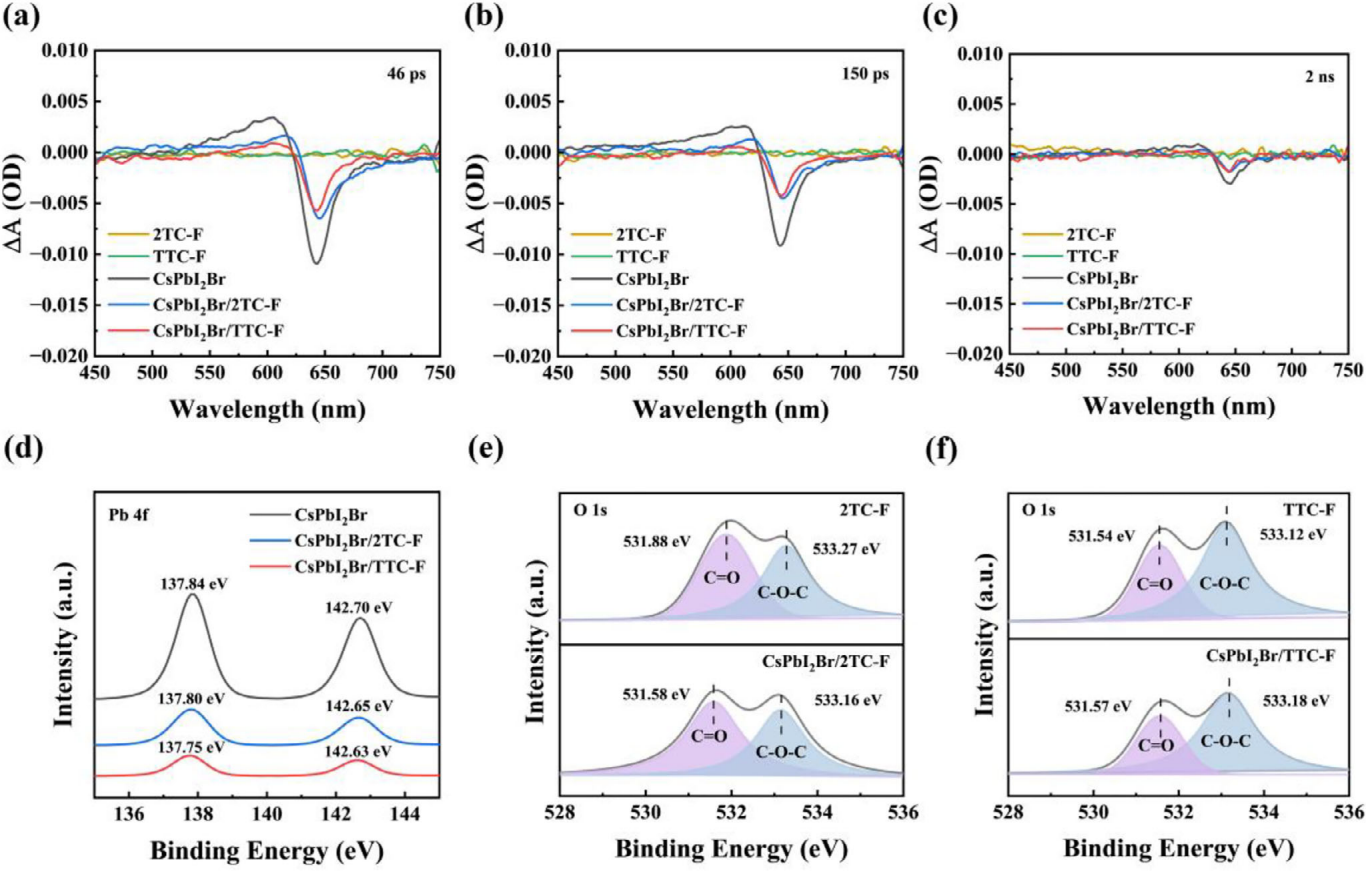
Transient absorption spectra of 2TC‐F, TTC‐F, CsPbI_2_Br, CsPbI_2_Br /2TC‐F, and CsPbI_2_Br /TTC‐F films at delay times of (a) 46 ps, (b) 150 ps, and (c) 2 ns, respectively; XPS spectra of (d) Pb 4f in CsPbI_2_Br, CsPbI_2_Br /2TC‐F, and CsPbI_2_Br /TTC‐F films; (e) O 1s in 2TC‐F and CsPbI_2_Br r/2TC‐F films; (f) O 1s in TTC‐F and CsPbI_2_Br /TTC‐F films.

To probe the charge transfer dynamics at the CsPbI_2_Br/HTL interface, the transient absorption spectroscopy (TAS) of HTLs, CsPbI_2_Br, and CsPbI_2_Br/HTLs was carried out. As shown in Figure 3a–c and Figure S5, the ground state bleach signal of CsPbI_2_Br is centered near 640 nm, corresponding to a negative absorption change (‐△A). At delay times of 46 ps, 150 ps, and 2 ns, both CsPbI_2_Br/2TC‐F and CsPbI_2_Br/TTC‐F films exhibit significantly weaker photobleaching signal intensities compared to the CsPbI_2_Br film, with a more rapid decay, indicating that holes were quickly transferred from the CsPbI_2_Br layer to the HTL. We further extracted the decay time constants τ_1_, τ_2_, and the average decay lifetime (τ_avg_) by performing a bi‐exponential fitting of the kinetic curve at 640 nm (Figure S6 and Table S4). In the CsPbI_2_Br/HTL system, τ_1_ typically corresponds to the process in which conduction band electrons are captured by trap states, whereas τ_2_ reflects the effective extraction of holes from CsPbI_2_Br to the HTL [46]. The fitting results indicate that both τ_2_ and τ_avg_ of the CsPbI_2_Br/TTC‐F film are significantly shortened, suggesting faster charge transfer between TTC‐F and CsPbI_2_Br, which benefits from their good energy level alignment and interface contact [47].

**FIGURE 4:**
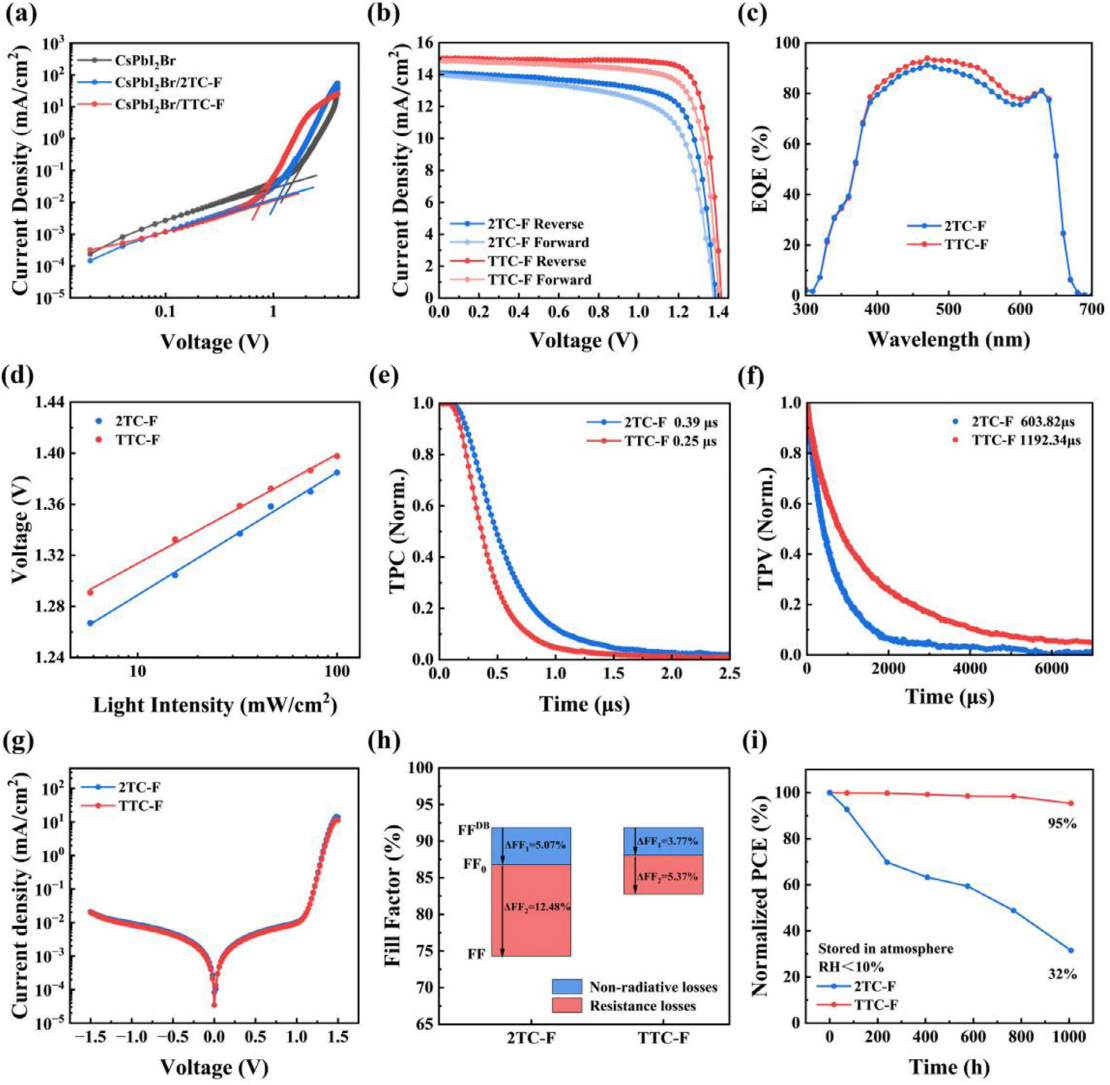
(a) Dark *J–V* curves of devices with the structure of glass/ITO/SnO_2_/CsPbI_2_Br/ (with or without ultrathin HTL)/C_60_/BCP/Ag; (b) *J–V* curves; (c) EQE curves; (d) *V*_OC_ vs. light intensity; (e) TPC measurement; (f) TPV measurement; (g) Dark current curves; (h) FF loss; (i) Normalized PCE evolution under low‐humidity conditions of CsPbI_2_Br PSCs based on 2TC‐F and TTC‐F HTMs.

In addition to extracting and transporting holes, HTL may also help passivate surface defects of perovskite. As shown in the electrostatic potential (ESP) mapping of 2TC‐F and TTC‐F (Figure S1), the negative potential is mainly localized on the oxygen atoms of the carboxylate groups, suggesting their possible coordination with under‐coordinated Pb^2+^ at the perovskite surface to passivate interfacial defects. To validate this interaction, X‐ray photoelectron spectroscopy (XPS) analysis was carried out. The results (Figure 3d–f; Figure S7) indicate that, compared with the CsPbI_2_Br thin film, the Pb 4f, Cs 3d, and I 3d peaks in both CsPbI_2_Br/2TC‐F and CsPbI_2_Br/TTC‐F films shifted toward lower binding energies. In comparison to the HTL, the O 1s, F 1s, and S 2p peaks in the CsPbI_2_Br/TTC‐F film shifted to higher binding energies, whereas in the CsPbI_2_Br/2TC‐F film, only an increase in the binding energy of the S 2p peak was observed. The interaction between HTM and CsPbI_2_Br relies on electron donor‐acceptor interactions; specifically, atoms in the HTM that are rich in lone pair electrons supply electrons to the perovskite layer, resulting in a reduction of their own electron cloud density and consequently shifting the XPS signal peak toward higher binding energies [24, 48, 49]. The above results indicate that due to the twisted molecular structure of 2TC‐F, its coordination mainly relies on the S atom; whereas TTC‐F, with its superior planarity, is able to form coordination bonds with perovskite surface ions through multiple sites involving O, S, and F, thereby exhibiting stronger passivation capabilities. This approach not only enhances the binding strength between the HTL and perovskite but also helps to suppress the nonradiative recombination losses caused by surface defect states [50]. Then, the trap state density (n_t_) was characterized for CsPbI_2_Br and HTM‐treated CsPbI_2_Br films using the SCLC method (Figure 4a). The n_t_ is calculated according to n_t_ =  2εε_0_V_TEL_/(eL^2^), where L represents the thickness of the perovskite layer, e is the elementary charge, V_TEL_ is the trap‐filled limit voltage corresponding to the intersection between the ohmic and trap‐filled regions, ε_0 _is the vacuum permittivity, and ε is the relative permittivity of CsPbI_2_Br. The V_TEL_ values of CsPbI_2_Br, CsPbI_2_Br/2TC‐F, and CsPbI_2_Br/TTC‐F are 1.50, 1.12, and 0.75 V, respectively, yielding n_t_ of 1.88 × 10^16^, 1.41 × 10^16^, and 9.41 × 10^15^ cm^−3^, respectively. The lowest n_t_ achieved with TTC‐F‐treated confirms its effective surface passivation.

We fabricated n‐i‐p structured CsPbI_2_Br PSCs with the architecture ITO/SnO_2_/CsPbI_2_Br/HTL/MoO_3_/Ag. We first optimized the thickness of the HTL by spin‐coating HTM solutions of different concentrations. The devices realized the best performance when the concentrations of 2TC‐F and TTC‐F were 8 and 10 mg mL^−1^, respectively (Figure S8 and Table S5). The current density‐voltage (*J–V*) curves of best‐performance devices under forward and reverse scans are shown in Figure 4b, with detailed photovoltaic parameters summarized in Table 1. The results indicate that, with the altered carboxylate group linkage site, the device employing TTC‐F as HTM achieved a PCE of 17.58% under reverse scan, with a *V*_OC_ of 1.415 V, a short‐circuit current density (*J*_SC_) of 15.01 mA cm^−2^, and a fill factor (FF) of 82.77%. This represents a significant enhancement compared to the 2TC‐F‐based device (PCE = 14.54%, *V*_OC_ = 1.386 V, *J*_SC_ = 14.12 mA cm^−2^, FF = 74.30%). Under forward scanning, the devices based on 2TC‐F and TTC‐F exhibit PCEs of 13.08% and 16.20%, respectively. The external quantum efficiency (EQE) curves (Figure 4c) yielded integrated current densities (*J*_int_) of 13.84 and 14.29 mA cm^−2^ for the devices based on 2TC‐F and TTC‐F, respectively, which are consistent with *J*_SC_ values from *J–V*. The reduction in EQE across the wavelength of 500–600 nm is ascribed to the strong intrinsic absorption of HTMs in this spectral region, which compromises the secondary light‑harvesting of the CsPbI_2_Br layer [33]. Statistical analysis of photovoltaic parameters (Figure S9) confirms that TTC‐F‐based devices exhibit superior *J*_SC_ and FF over 2TC‐F‐based devices, along with higher overall performance. The TTC‐F‐based CsPbI_2_Br PSCs also demonstrated significantly superior photovoltaic performance compared with the CsPbI_2_Br PSCs using PTAA and Spiro‐OMeTAD as HTLs, which delivered PCEs of 14.14% and 15.74%, respectively (Figure S10 and Table S6). This enhancement is attributed to the deeper HOMO level, more ordered molecular stacking, and effective surface defect passivation, which collectively enhance charge extraction and transport.

**TABLE 1:** Photovoltaic parameters of single‐junction CsPbI_2_Br PSCs.

HTM	Scan	*V*_OC_ (V)	*J*_SC_ (mA cm^−2^)	FF (%)	PCE (%)
2TC‐F	Reverse	1.386	14.12	74.30	14.54
	Forward	1.378	14.00	67.81	13.08
TTC‐F	Reverse	1.415	15.01	82.77	17.58
	Forward	1.402	14.88	77.65	16.20

To investigate the recombination behavior of charge carriers within the device, the variation of *V*_OC_ under different light intensities (P_light_) was recorded, and the ideality factor (n) was calculated using the equation *V*_OC_ =  nk_B_Tln(P_light_)/q + B. The n values of the devices based on 2TC‐F and TTC‐F were determined to be 1.61 and 1.44, respectively (Figure 4d). The relatively lower n‐value of the TTC‐F‐based device suggests significantly suppressed trap‐assisted recombination [51]. Transient photovoltage (TPV) and transient photocurrent (TPC) measurements were further employed to analyze charge extraction and recombination dynamics (Figure 4e,f). The TTC‐F‐based device exhibits a shorter photovoltage decay lifetime (0.25 vs. 0.39 µs) and a longer photocurrent decay lifetime (1192.34 vs. 603.82 µs), indicating an enhanced carrier collection efficiency and reduced interfacial non‐radiative recombination, which contribute to higher FF and *J*_SC_. Dark *J–V* measurements (Figure 4g) reveal a lower leakage current in the TTC‐F device compared to the 2TC‐F counterpart, indicating reduced shunting paths and better charge collection, thereby supporting the higher *J*_SC_ [52]. Electrochemical impedance spectroscopy (EIS) was also conducted, and the Nyquist plots (Figure S11) show a larger recombination resistance for the TTC‐F device, which helps suppress non‐radiative recombination and thus enhances *V*_OC_ [53].

To understand the origin of the FF difference between the two devices, we performed an analysis of the FF losses (Figure 4h and Table S7). FF losses are divided into nonradiative losses (Δ FF_1_ = FF^DB^ ‐FF_0_) and charge transport losses (Δ FF_2_ = FF_0_ ‐FF), the latter primarily resulting from resistive effects such as series and parallel resistances [54]. For CsPbI_2_Br PSCs, the theoretical FF^DB^ is 91.85% [36]. FF_0_, which represents the maximum fill factor in the absence of charge transport losses, can be calculated using the formula FF_0_ =  [(q*V*_OC_)/(nk_B_T)‐ln(q*V*_OC_)/(nk_B_T) + 0.72)][(q*V*_OC_/(nk_B_T) + 1] [2]. The calculated FF_0_ values of the TTC‐F and 2TC‐F‐based devices are 88.08% and 86.78%, respectively. Their transmission losses, ΔFF_1_, are 3.77% and 5.07%, respectively, while the ΔFF_2_ values related to nonradiative recombination are 5.31% and 12.48%, respectively. This result indicates that the TTC‐F device significantly reduces charge transport losses, which is closely related to its higher conductivity, more ordered molecular stacking, and lower resistance, thereby facilitating more efficient carrier transport toward the electrodes.

In addition to superior photovoltaic performance, TTC‐F‐based devices also exhibit excellent environmental stability. We compared the stability of devices based on 2TC‐F and TTC‐F after aging for 1000 h in a nitrogen glovebox and in an atmospheric environment with relative humidity below 10% (under indoor ambient light, temperature 15–25°C). After 1000 h of aging in an N_2_ glovebox, the TTC‐F‐based devices retained 97% of their initial PCE, whereas the 2TC‐F‐based devices maintained only 57%. When stored at <10% relative humidity for 1000 h, the PCE of TTC‐F‐based devices remained at 95%, significantly higher than the 32% observed for 2TC‐F‐based devices (Figure 4i). Additionally, under continuous thermal stress at 60°C for 1000 h, the TTC‐F‐based devices also showed markedly better stability, retaining 94% of the initial PCE, whereas the 2TC‐F control degraded to 23% (Figure S12).

**FIGURE 5:**
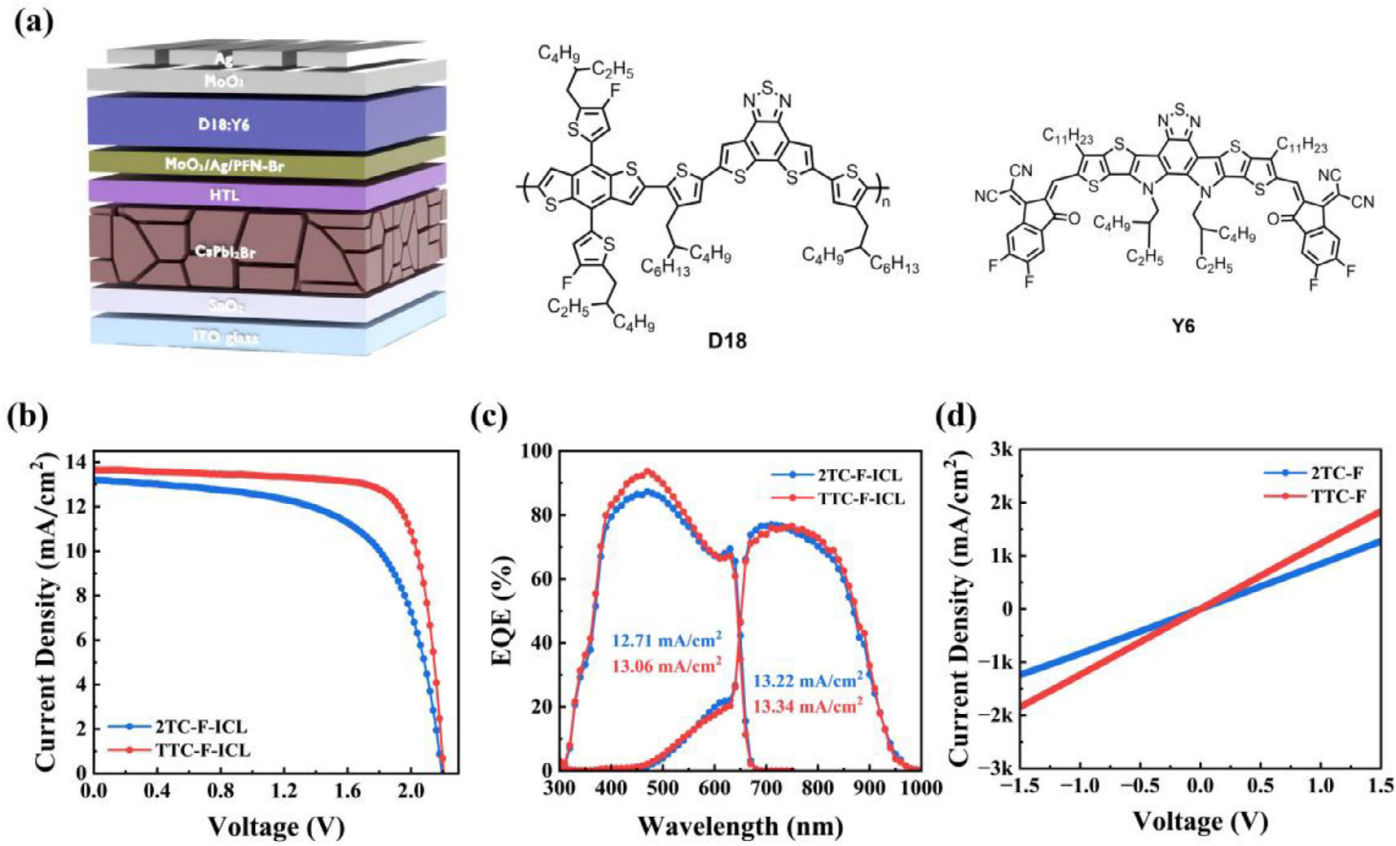
(a) Device structure of TSC and molecular structures of D18 and Y6; (b) *J–V* and EQE curves of TSCs based on 2TC‐F‐ICL or TTC‐F‐ICL; (c) *J–V* curves of ITO/ICL/Ag devices.

We also fabricated a perovskite/organic TSC using CsPbI_2_Br as the front sub‐cell, D18:Y6 as the rear sub‐cell, and 2TC‐F (or TTC‐F)/MoO_3_/Ag/PFN‐Br as ICL (Figure 5a). The D18:Y6‐based single‐junction organic solar cell achieved a PCE of 17.08% (*V*_OC_ = 0.857 V, *J*_SC_ = 26.15 mA cm^−2^, FF = 76.20%) (Figure S13 and Table S8). The *J–V* curves of TSCs are presented in Figure 5b (for detailed photovoltaic parameters, see Table S9). The TTC‐F‐based TSCs achieved a maximum PCE of 23.29%, with a *J*_SC_ of 13.66 mA cm^−2^, a *V*_OC_ of 2.21 V, and an FF of 77.27%. The 2TC‐F‐based TSCs yielded a PCE of 18.17%, with a *V*_OC_ of 2.20 V, a *J*_SC_ of 13.10 mA cm^−2^, and an FF of 63.20%. Figure 5c shows the EQE curves of the TSCs based on 2TC‐F and TTC‐F. The *J*_int_ of both sub‐cells align well with the *J*_SC_ values obtained from *J–V* measurements, confirming excellent current matching. To analyze the interfacial contact characteristics of different HTMs within the ICL, we constructed ICL test devices with the structure ITO/HTL/MoO_3_/Ag/PFN‐Br/Ag, denoted as TTC‐F‐ICL and 2TC‐F‐ICL, respectively. As shown in Figure 5d, both TTC‐F‐ICL and 2TC‐F‐ICL exhibit symmetric and nearly linear *J–V* characteristics, demonstrating the formation of quasi‐Ohmic contact and lower interlayer contact barriers within the ICL. The steeper slope of the *J–V* curve for TTC‐F‐ICL further reveals lower resistive loss in the corresponding ICL, which contributes to the enhanced performance of the TSCs.

## 3 Conclusion

In summary, this work demonstrates that the positioning of carboxylate groups in conjugated polymers profoundly influences their optoelectronic properties and device performance. By comparing isomeric polymers 2TC‐F (carboxylate on T unit) and TTC‐F (carboxylate on TT unit), we establish that the carboxylate group on the TT unit in TTC‐F yields a deeper HOMO energy level (−5.64 eV), which matches the perovskite valence band better and promotes a more planar main chain conformation. These features enhance *π–π* stacking, improve film morphology, and increase hole mobility. Moreover, TTC‐F exhibits superior defect passivation capability of the perovskite surface, thereby suppressing non‐radiative recombination more effectively than 2TC‐F. As a result, TTC‐F‐based CsPbI_2_Br PSCs achieved a PCE of 17.58%, a 21% improvement over 2TC‐F‐based devices (14.54%). The TTC‐F‐based devices also show outstanding stability under thermal, low‐humidity, and inert‐atmosphere aging, far exceeding the 2TC‐F counterparts. When integrated as a hole‐transport component in ICL for CsPbI_2_Br/D18:Y6 TSCs, TTC‐F enabled a remarkable PCE of 23.29%. This study highlights the crucial role of site‐specific carboxylate functionalization in modulating molecular packing, charge transport, and interfacial properties, offering a rational design strategy for developing highly efficient and stable HTMs.

## Conflicts of Interest

The authors declare no conflict of interest.

## Supporting information




**Supporting File**: advs74111‐sup‐0001‐SuppMat.docx.

## Data Availability

The data that support the findings of this study are available on request from the corresponding author. The data are not publicly available due to privacy or ethical restrictions.
