# Supplementary material for: Site‐Specific Carboxylate Functionalization in Conjugated Hole Transport Polymers Enables Efficient CsPbI2Br Single‐Junction and Tandem Solar Cells
Source: Adv Sci (Weinh). 2026 Feb 15;13(19):e24013. doi: 10.1002/advs.202524013 (PMC13045486; doi:10.1002/advs.202524013)
Supplement: Supplementary file 1 — Supporting File: advs74111‐sup‐0001‐SuppMat.docx. [file ADVS-13-e24013-s001.docx]

Supporting Information

**Site-Specific Carboxylate Functionalization in Conjugated Hole Transport Polymers Enables Efficient CsPbI_2_Br Single-junction and Tandem Solar Cells**

*Jingfei Wang, Weilin Zhang, Haobin Zhang, Zongwei Chen, Tangyue Xue, Qing Guo, Zhi Zheng*, Zongtao Wang, Erjun Zhou*, Qiang Guo**

J. Wang, W. Zhang, H Zhang, Z Chen, T Xue, Q. Guo, Dr. Q. Guo*

Henan Institute of Advanced Technology, Zhengzhou University, Zhengzhou 450003, China.

E-mail: [guoqiang@zzu.edu.cn](mailto:guoqiang@zzu.edu.cn)

Z. Wang, Prof. E. Zhou*

College of Biological and Chemical Engineering, Jiaxing University, Jiaxing 314001, China.

E-mail: zhouej@nanoctr.cn

Prof. Z. Zheng

College of Chemical and Materials Engineering, Xuchang University, Xuchang, Henan 461000, China

E-mail: zzheng@xcu.edu.cn

**Experimental Section**

**Materials:** HPbI_3_, HPbBr_3_, Spiro-OMeTAD, **Poly[bis(4-phenyl)(2,4,6-trimethylphenyl)amine]** (PTAA), and Tris(2-(1H-pyrazol-1-yl)-4-tert-butylpyridine)cobalt(III)tris(bis(trifluoromethylsulfonyl)imide) (Co-TFSI) were purchased from Xi’an Yuri Solar Co., Ltd.; HCOOCs·H_2_O from Maclin Biochemical Technology Corp; SnO_2_ colloidal dispersion (15% in H_2_O) from Alfa Aesar; Lithium bis(trifluoromethanesulfonyl)imide (Li-TFSI) and 4-tert-Butylpyridine (TBP) from Advanced Election Technology Co., Ltd.; D18 and Y6 from Hyper Inc; PFN-Br from Solarmer Materials Inc; chloroform (CF) from Tianjin Kemiou Chemical Reagent Co., Ltd.; chlorobenzene (CB) from Thermo Fisher Scientific (China) Co., Ltd.; Acetonitrile from Beijing InnoChem Science & Technology Co., Ltd.; ultra-dry dimethyl sulfoxide (DMSO) from Acros Organics. The hole-transport polymers 2TC-F and TTC-F were synthesized following our reported method.^[1]^

**Precursor preparation:** The SnO_2_ precursor was obtained by diluting the commercial tin(IV) oxide colloidal dispersion (15% in H_2_O) twofold with ultrapure water under overnight stirring. The CsPbI_2_Br perovskite precursor (1.2 M) was formulated by dissolving HPbI_3_ (471.1 mg), HPbBr_3_ (179.6 mg), and HCOOCs·H_2_O (235.2 mg) in DMSO, followed by overnight stirring at 60 °C within a glovebox. For the hole transport materials (HTMs) solution, 2TC-F (6-10 mg mL^-1^) and TTC-F (8-12 mg mL^-1^) were dissolved in CF, and PTAA (10 mg mL^-1^) was dissolved in CB. Spiro-OMeTAD solution was obtained by dissolving 72.3 mg spiro-OMeTAD in 1 mL of CB and added with 17.5 μL of Li-TFSI (520 mg mL^-1^ in acetonitrile), 29 μL of TBP, and 17.5 μL of Co-TFSI (300 mg mL^-1^ in acetonitrile). For the organic active layer solution, a D18:Y6 blend (1:1.6, w/w) was dissolved in CF at a concentration of 11 mg mL^-1^ and stirred at 80 °C for 2h before use. Lastly, a PFN-Br solution was prepared in methanol at 0.5 mg mL^-1^.

**Preparation of single-junction perovskite solar cells:** Following spin-coating and annealing (150 °C, 30 min) of the SnO_2_ precursor on ITO, the substrates were transferred to a nitrogen glovebox. The CsPbI_2_Br perovskite layer was then deposited via a two-step spin-coating (1000 rpm for 10 s; 2000 rpm for 30 s) and crystallized by sequential annealing at 60 °C and 250 °C for 10 min each. Subsequently, the HTM solutions were spin-coated (3000 rpm, 30 s) on CsPbI_2_Br film to form the hole transport layer (HTL). Device fabrication was completed by the sequential thermal evaporation of MoO_3_ (8 nm) and Ag (100 nm) electrodes.

**Fabrication of Tandem Solar Cells:** With the front cell HTL deposited, MoO_3_ (8 nm) and Ag (1 nm) were sequentially evaporated, followed by spin-coating a PFN-Br solution at 4000 rpm for 30 s. The organic photoactive film was then spin-coated from the D18:Y6 (1:1.6, w/w) solution (3000 rpm, 30 s) onto PFN-Br and annealed in a CF vapor atmosphere for 8 min. For CF vapor atmosphere, a total of 20 μL of CF was dispensed (split between both sides of the device), and the device was immediately covered with a 6 cm-diameter Petri dish to create an enclosed space. The setup was then maintained at 25 °C to allow the chloroform to evaporate naturally, thereby forming a uniform solvent atmosphere. Complete TSCs were obtained by final thermal evaporation of MoO_3_ (10 nm) and Ag (100 nm) electrodes.

**Preparation of** **single-junction organic solar cells:** A ZnO precursor was spin-coated onto pre-cleaned ITO at 4000 rpm for 30 s and annealed at 150 °C for 30 min. After transferring into glovebox, the D18:Y6 precursor solution was spin-coated on the ZnO layer at 3000 rpm for 30 s and annealed in chloroform vapor atmosphere for 8 min. Finally, MoO_3_ (10 nm) and Ag (100 nm) were thermally evaporated to complete the devices.

**Instrumentation and characterization:** Ultraviolet-visible absorption and transmission spectra were measured using a Shimadzu UV-3600 spectrophotometer. Cyclic voltammetry (CV) was conducted on an electrochemical workstation equipped with a standard three-electrode configuration. Grazing-incidence wide-angle X-ray scattering (GIWAXS) measurements were performed on an XEUSS SAXS/WAXS system, with data collected by a Pilatus 100k area detector (195×487 pixels, pixel size: 0.172 mm×0.172 mm), using an X-ray wavelength of 1.54 Å and an incident angle of 0.2°. In dark conditions，the dark current curve and conductivity were measured using the Zolix Solar IV-150A system. Atomic force microscopy (AFM) and Kelvin probe force microscopy (KPFM) analyses were conducted with the SPM-9700HTTM. Transient absorption spectroscopy (TAS) measurements were performed using a regenerative amplified Ti:sapphire laser system (Coherent) as the light source and a HELIOS spectrometer series (Ultrafast Systems LLC) for detection, with the excitation wavelength set to 380 nm. The CsPbI_2_Br and CsPbI_2_Br/HTL films for TAS measurements were deposited on quartz substrate. Additionally, transient photovoltage (TPV) and transient photocurrent (TPC) were evaluated in the dark using a solar cell characterization analyzer (PAIOS, Fluxim). The light intensity for TPC and TPV tests is 50.0% 1 sun and 0.1% 1sun, respectively. X-ray photoelectron spectroscopy (XPS) data were acquired using a Thermo Scientific K-Alpha+. Current density-voltage (*J-V*) curves were measured under an AM 1.5 G solar simulator inside a glovebox using a Keithley 2400 digital source meter, with a scanning speed of 150 mV s^-1^ for the *J-V* measurements. The effective area of the devices for the *J-V* tests was 5 mm^2^. External quantum efficiency (EQE) curves were measured using a Zolix SCS10-X150-DSSC system. For tandem solar cells, EQE measurements were conducted with spectral filter bias illumination on the device surface: the EQE of the front sub-cell (perovskite solar cell) was measured using an 880 nm laser, while that of the rear sub-cell (organic solar cell) was measured using a 460 nm laser.


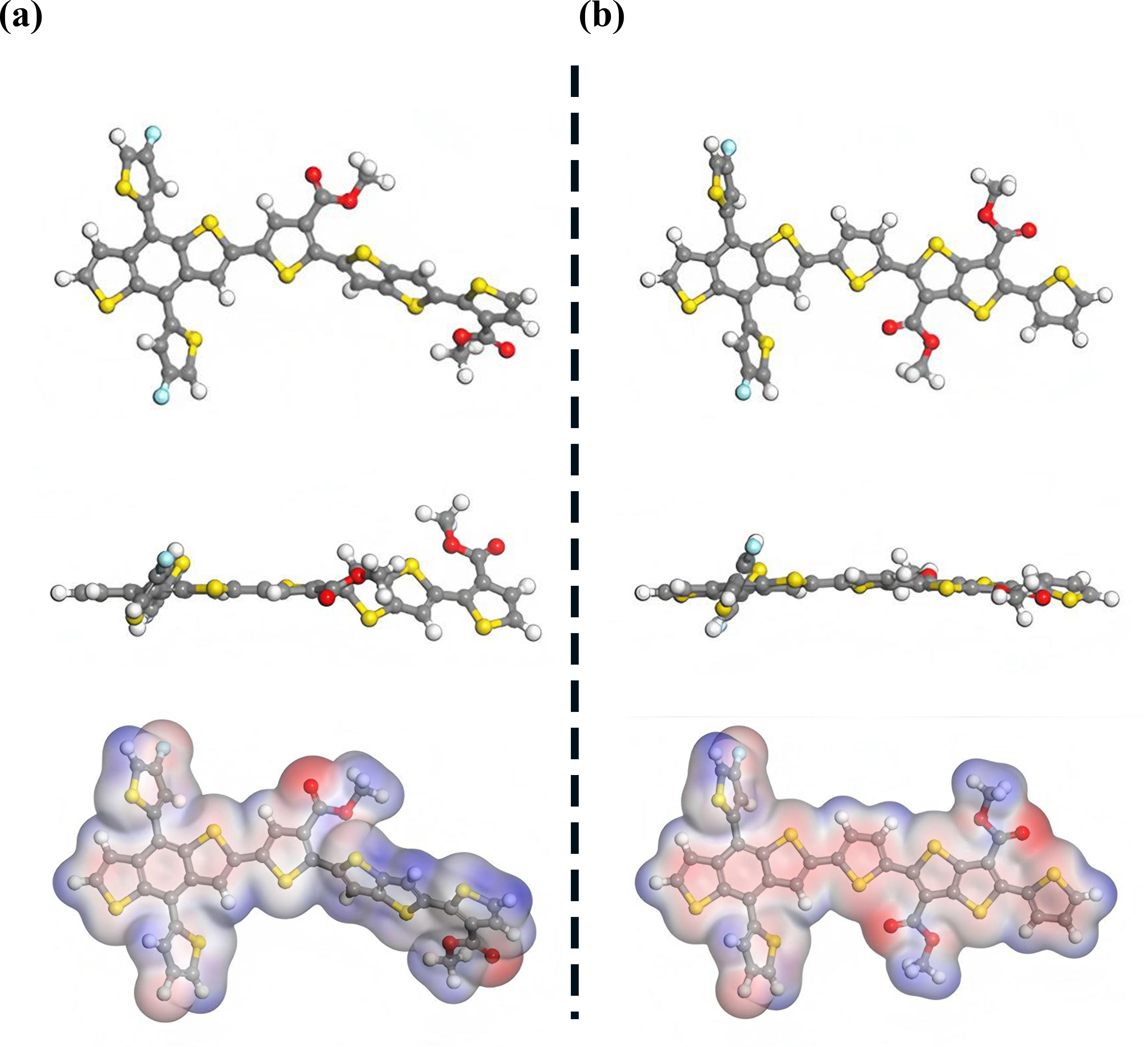


**Figure S1.** Top views, side views, and electrostatic potential maps of the structurally optimized repeat units of (a) 2TC-F and (b) TTC-F.


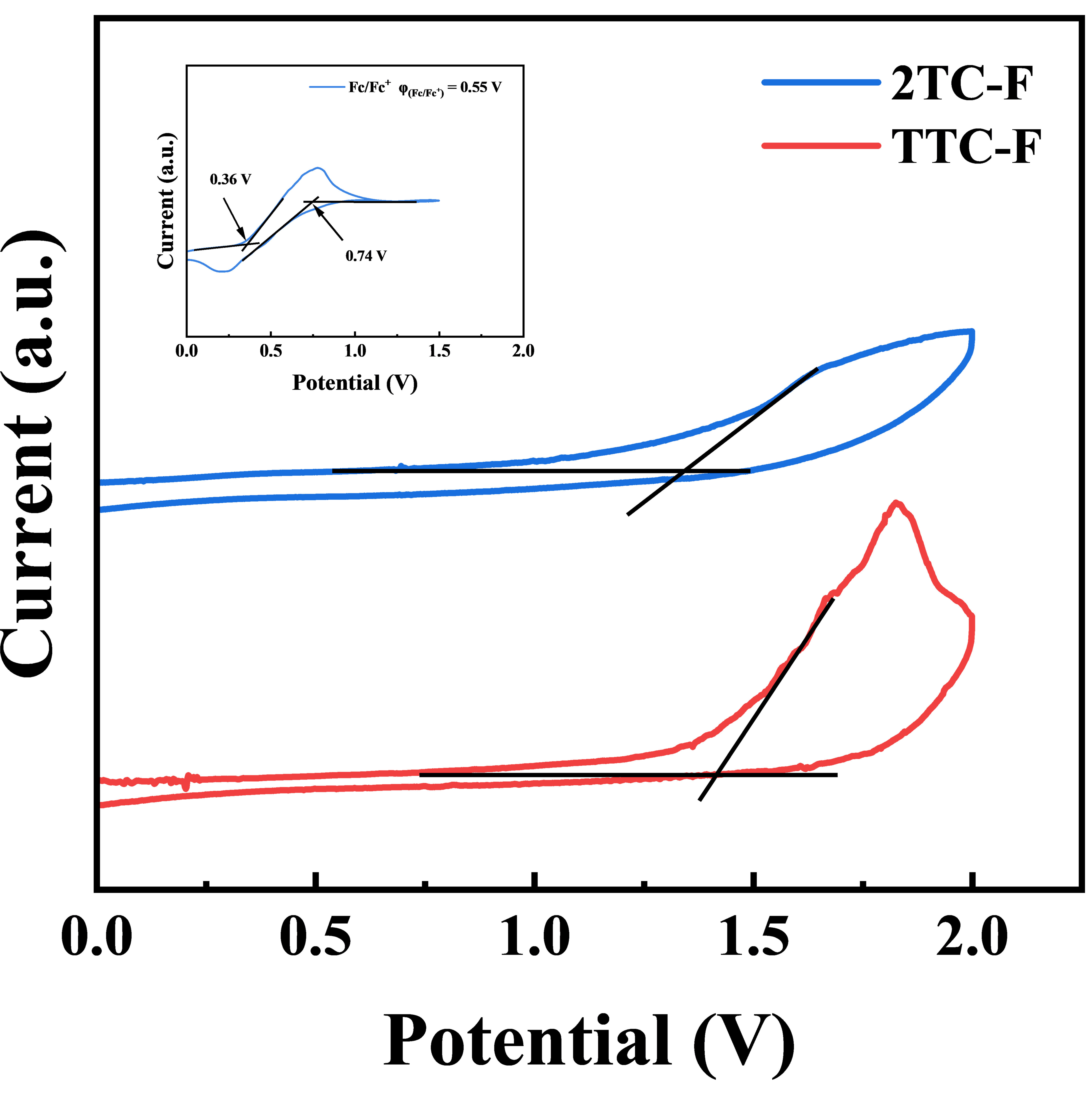


**Figure S2.** Cyclic voltammograms of 2TC-F, TTC-F, and Fc/Fc⁺ measured with Ag/AgCl as the reference electrode.


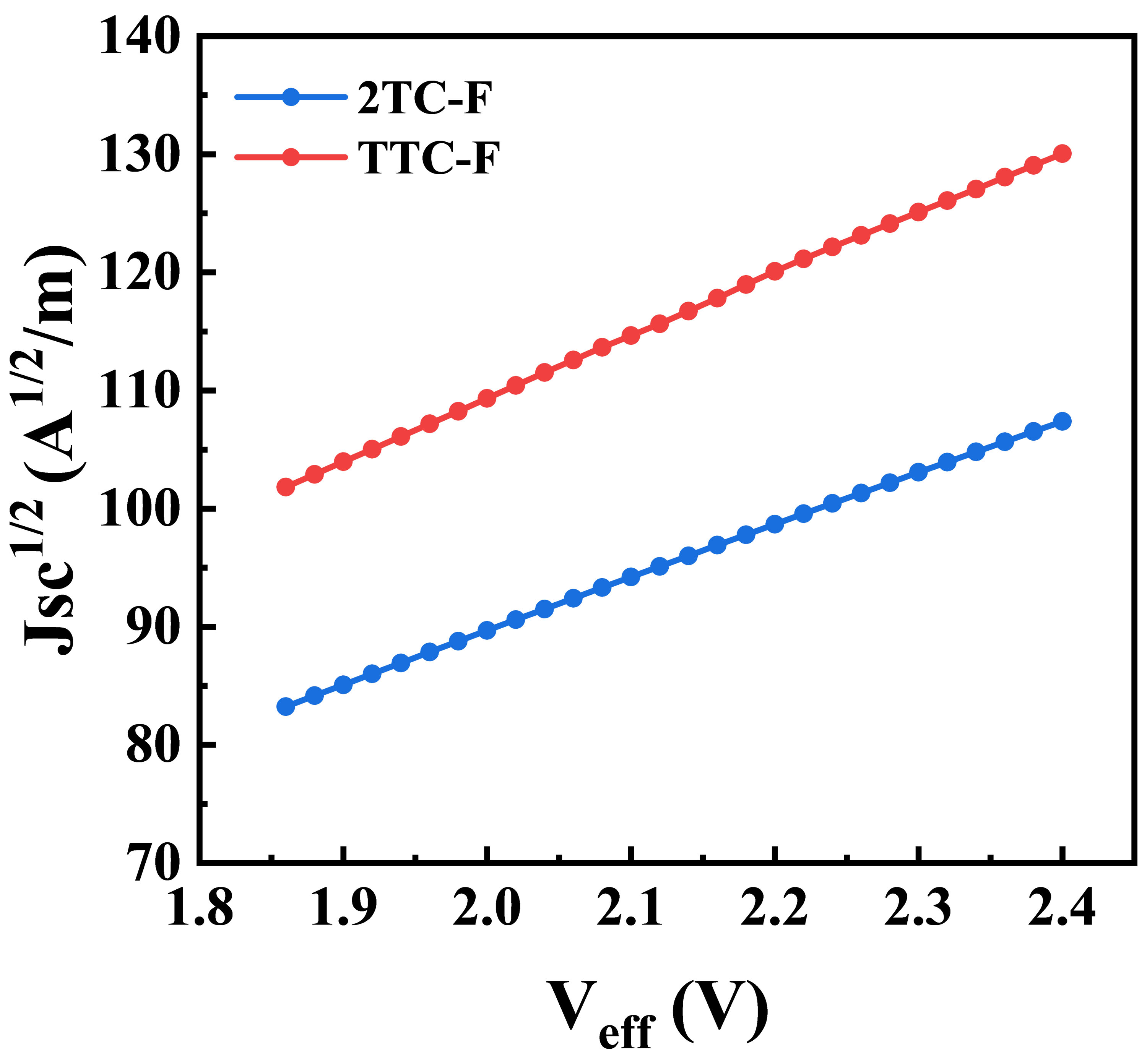


**Figure S3.** Jsc^1/2^-V curves of the ITO/PEDOT:PSS/HTM/Au device.


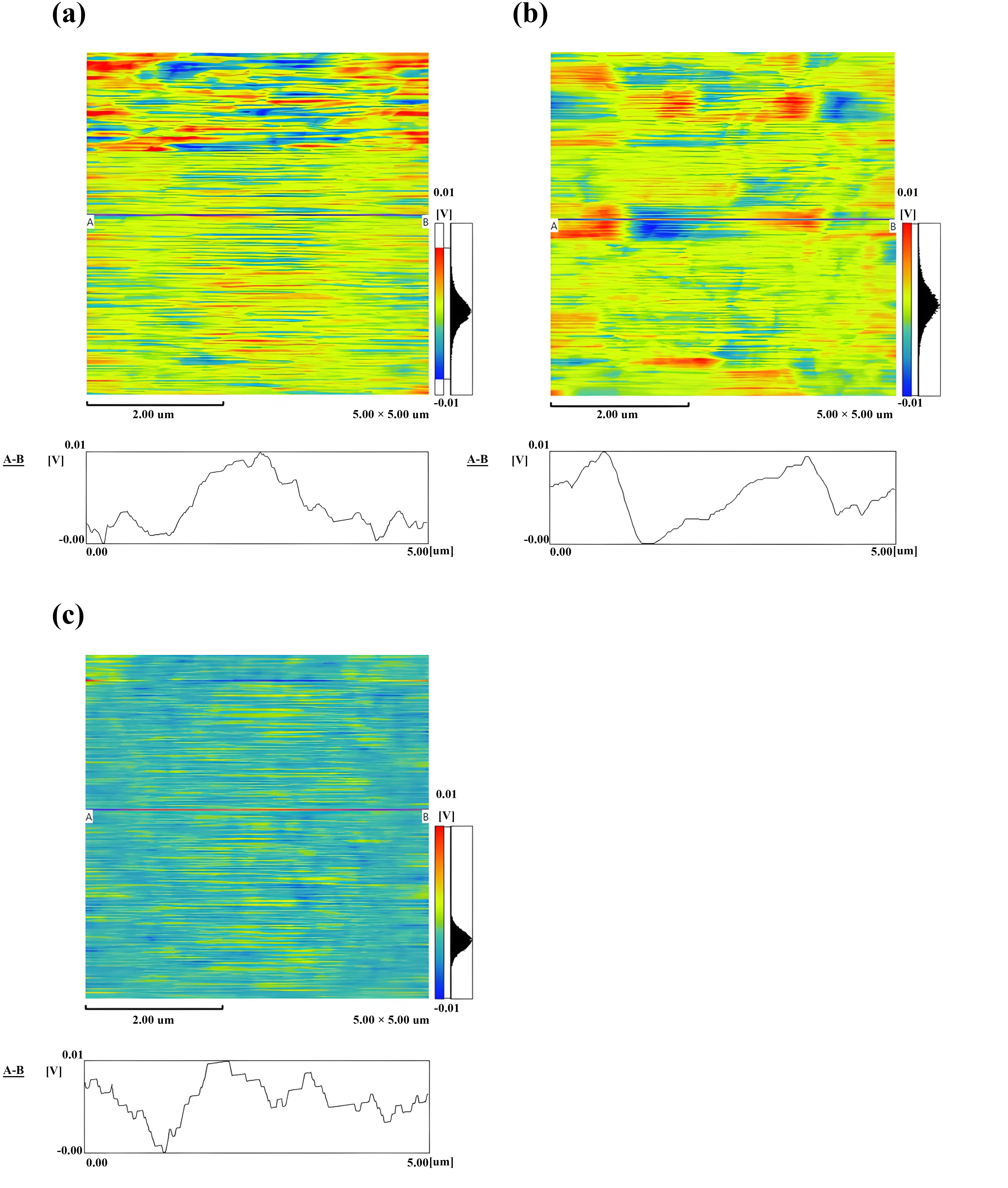


**Figure S4.** Surface potential distribution maps of (a) CsPbI_2_Br, (b) CsPbI_2_Br/2TC-F, and (c) CsPbI_2_Br/TTC-F samples.


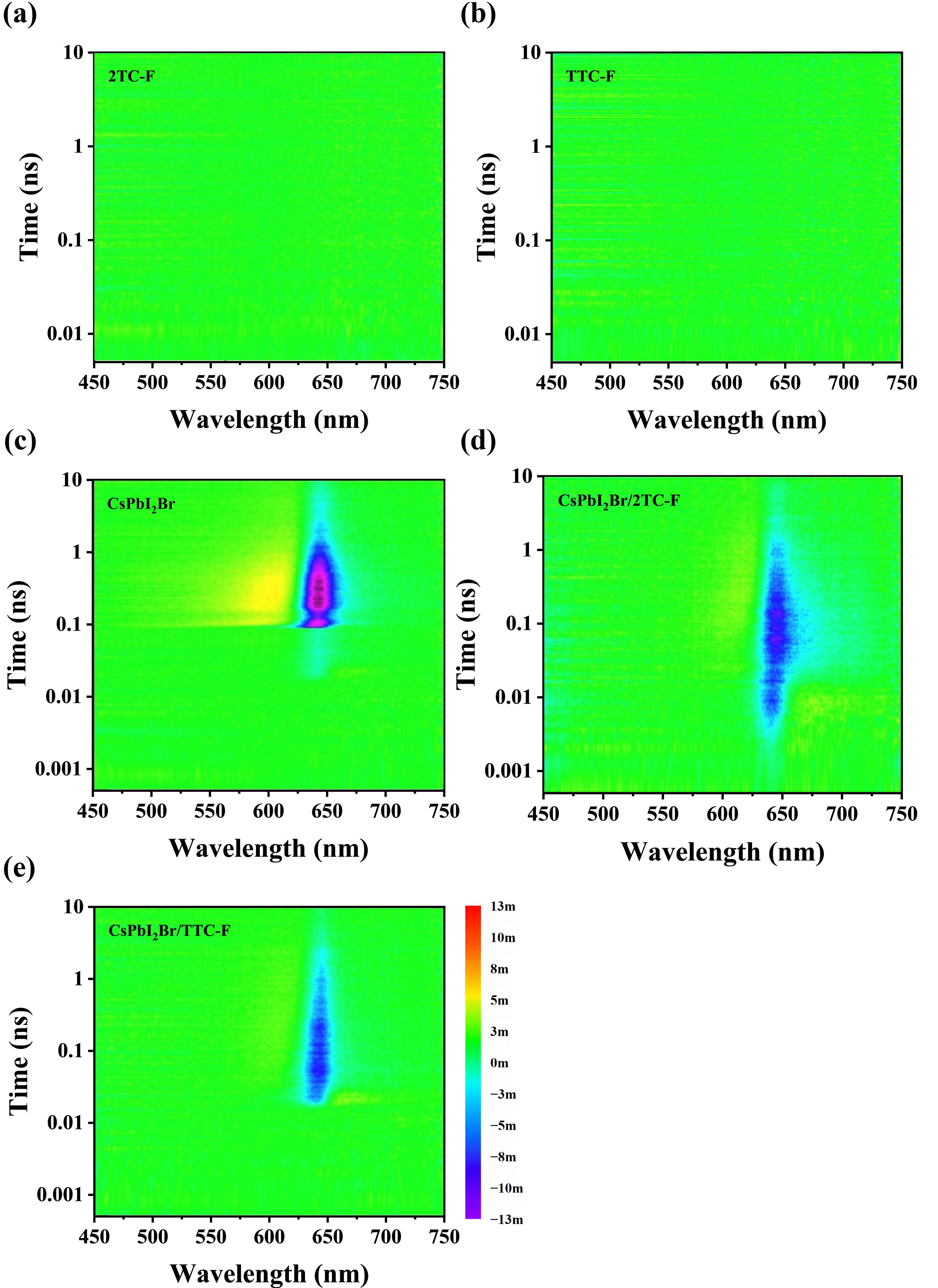


**Figure S5.** Two-dimensional transient absorption spectra maps of (a) 2TC-F, (b) TTC-F, (c) CsPbI_2_Br, (d) CsPbI_2_Br/2TC-F, and (e) CsPbI_2_Br/TTC-F samples.


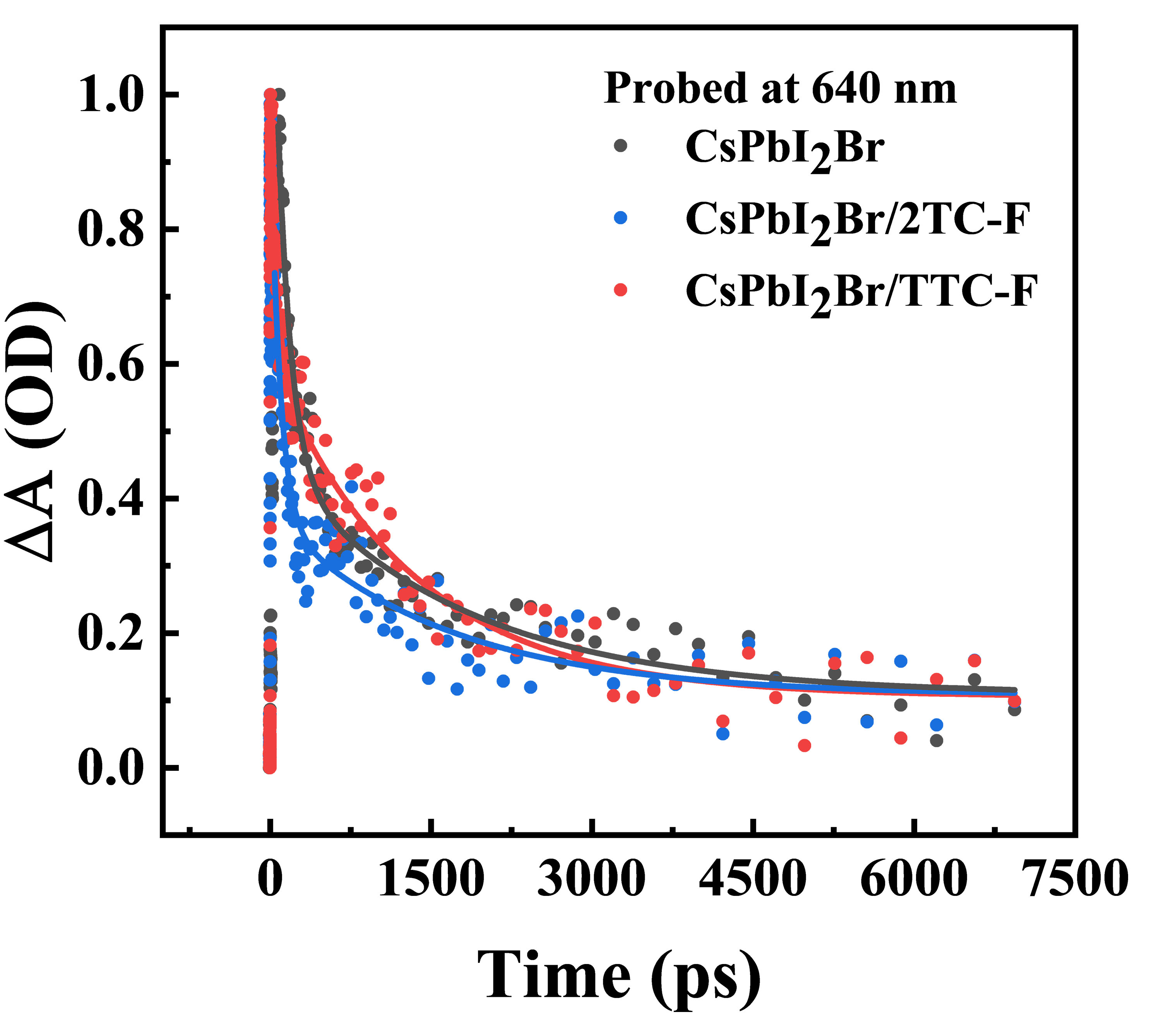


**Figure S6.** Kinetic traces of CsPbI_2_Br, CsPbI_2_Br/2TC-F, and CsPbI_2_Br/TTC-F samples.


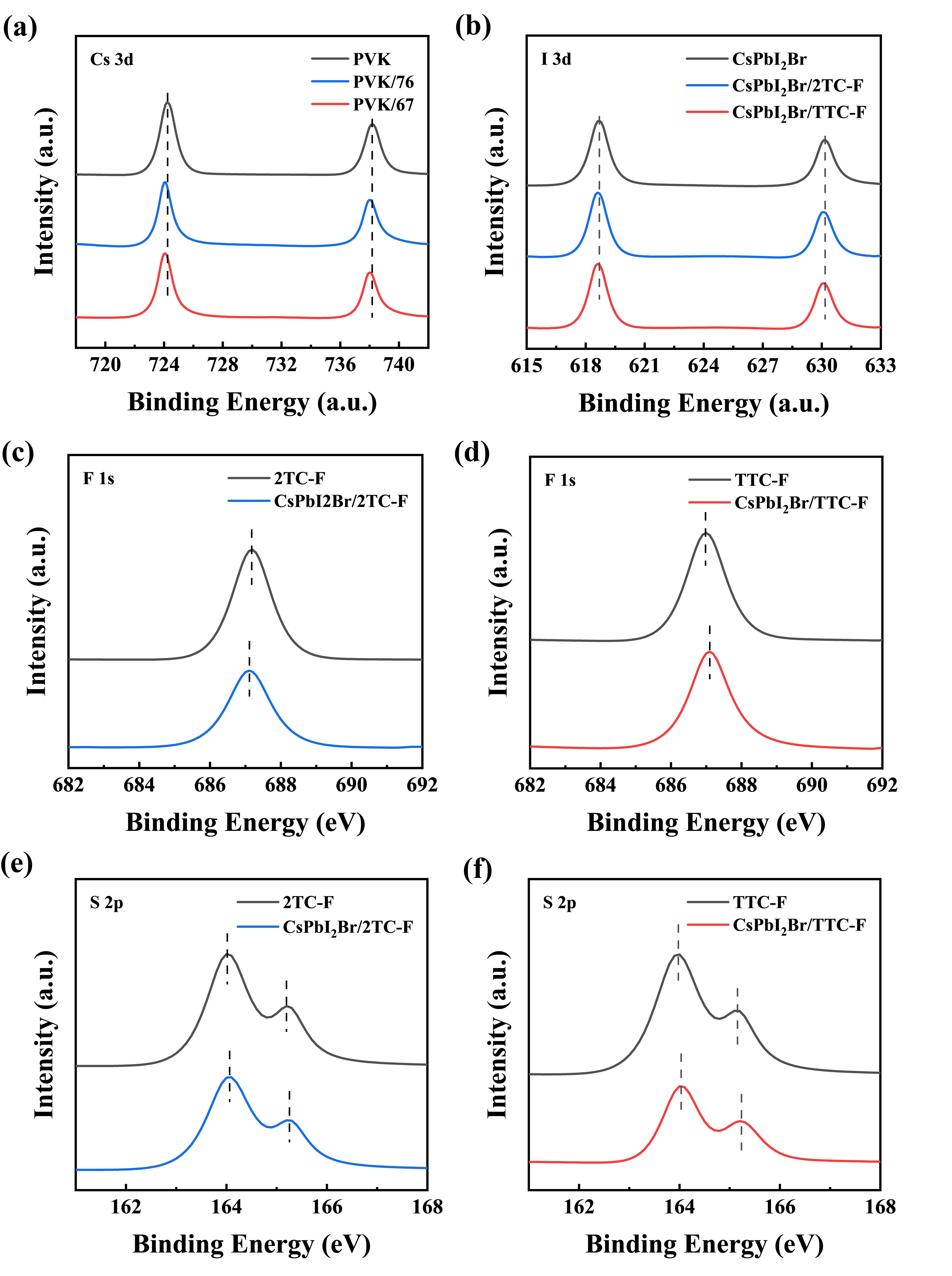


**Figure S7.** XPS spectra of (a) Cs 3d and (b) I 3d in CsPbI_2_Br, CsPbI_2_Br/2TC-F, and CsPbI_2_Br/TTC-F films; XPS spectra of (c) F 1s and (d) S 2p in 2TC-F and CsPbI_2_Br/2TC-F films; XPS spectra of (e) F 1s and (f) S 2p in TTC-F and CsPbI_2_Br/TTC-F films.


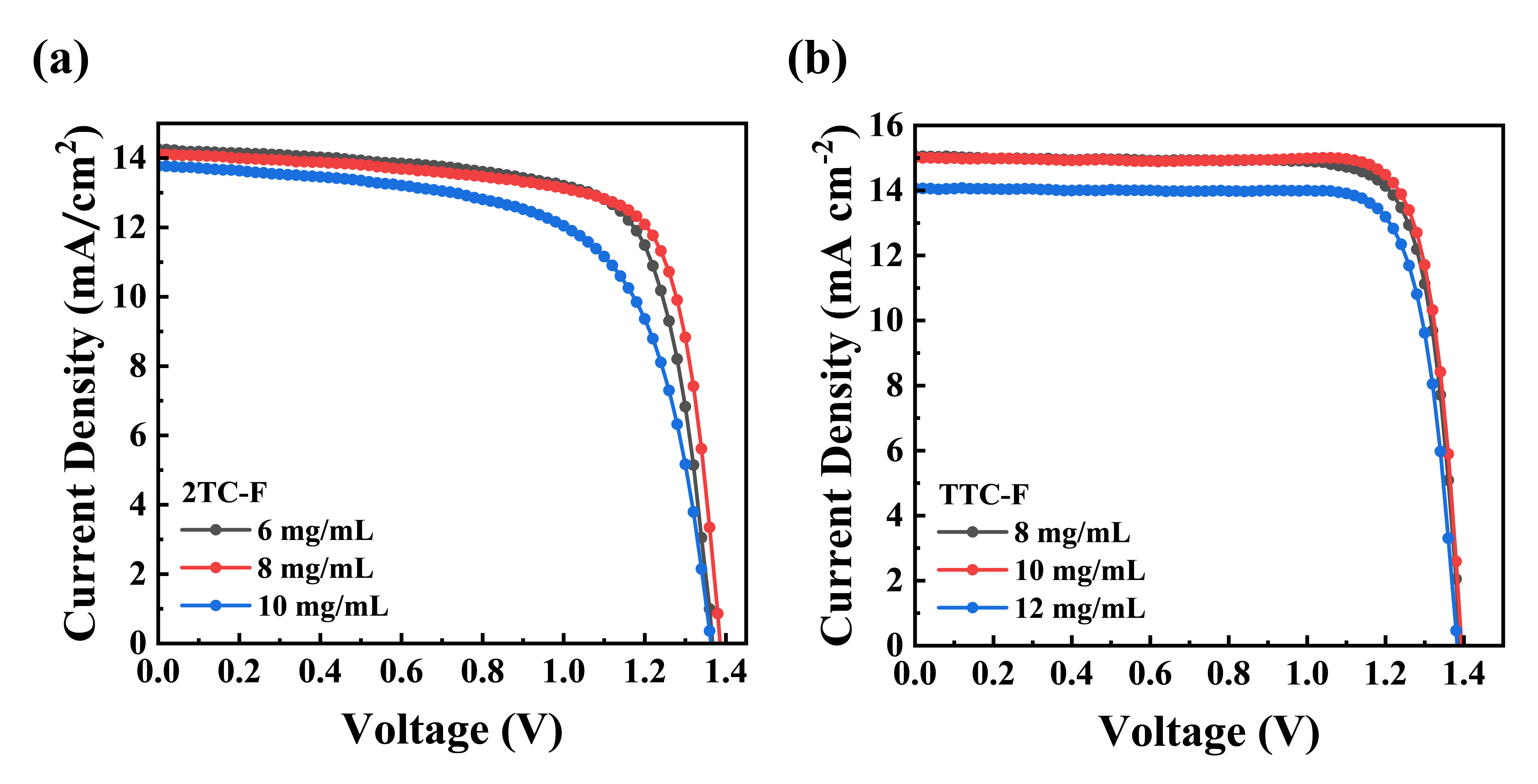


**Figure S8.** *J-V* curves of CsPbI_2_Br PSCs based on different concentrations of (a) 2TC-F and (b) TTC-F.


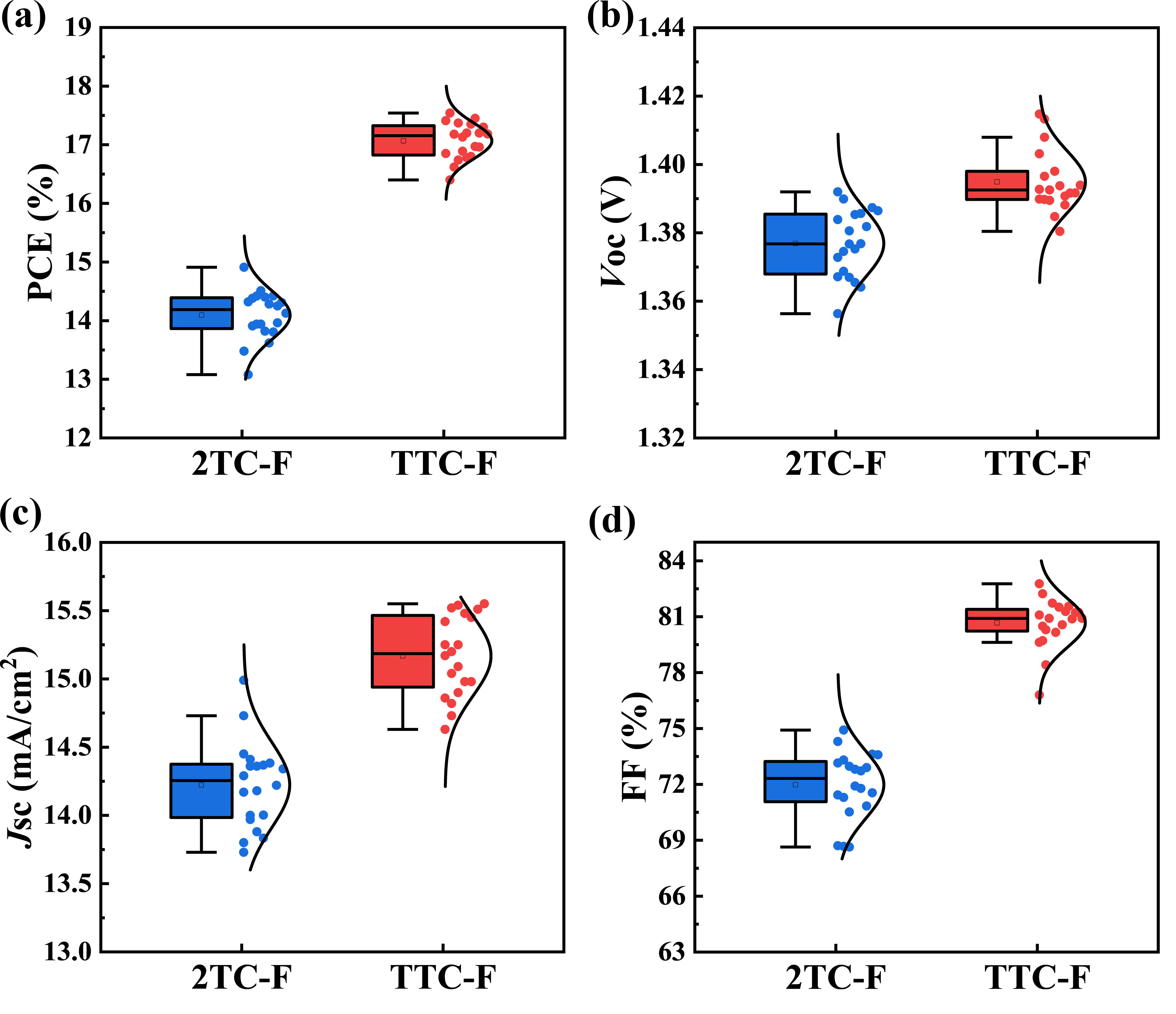


**Figure S9.** Statistical distributions of (a) PCE, (b) *V*_OC_, (c) *J*_SC_, and (d) FF for 20 independent CsPbI_2_Br PSCs based on 2TC-F and TTC-F HTMs.


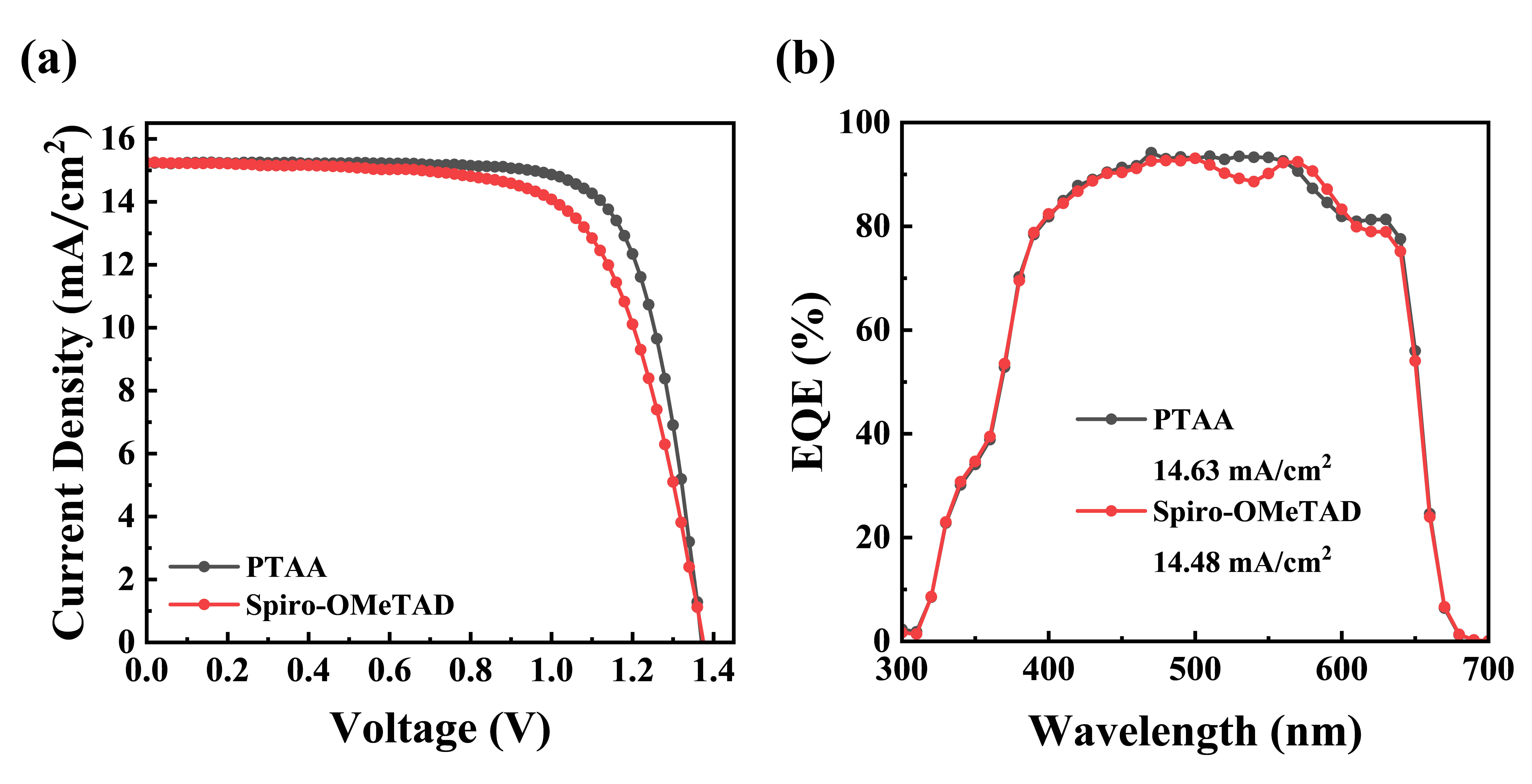


**Figure S10.** (a) *J-V* and (b) EQE curves of CsPbI_2_Br PSCs using PTAA or Spiro-OMeTAD as HTM.


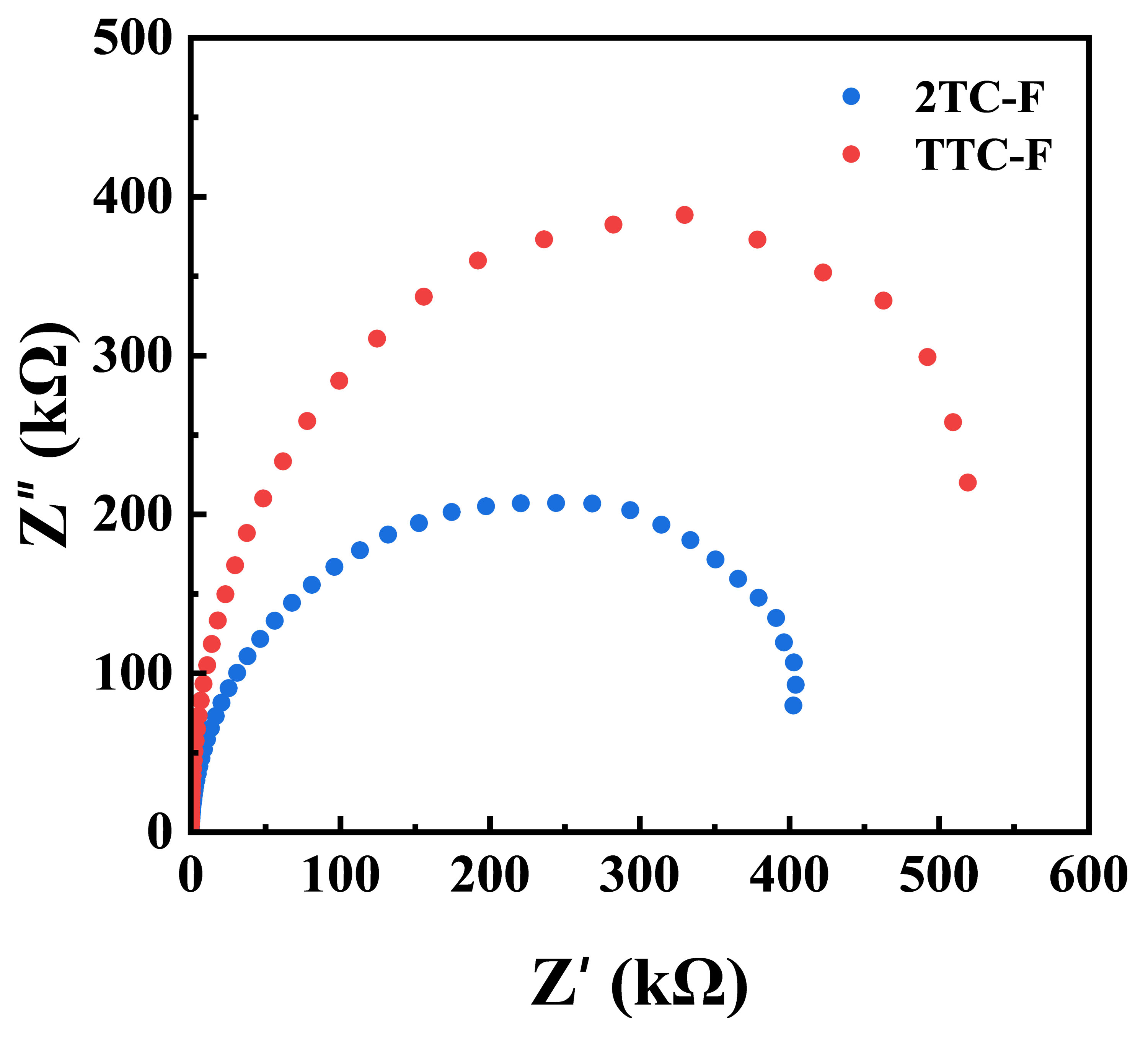


**Figure S11.** Nyquist plot of the device.


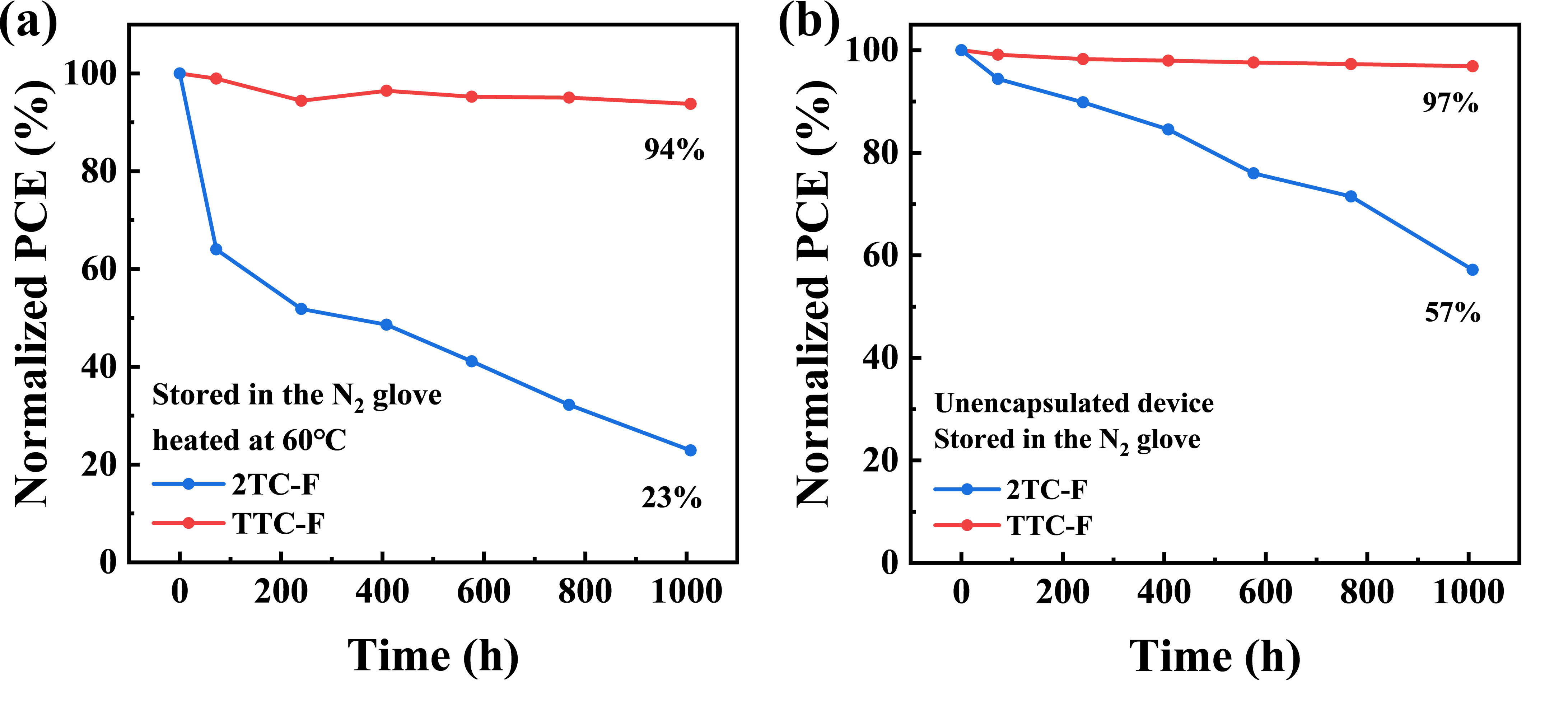


**Figure S12.** (a) PCE decay curves aged in a N_2_ glovebox and (b) PCE decay curves aged under continuous heating at 60°C in a glovebox.


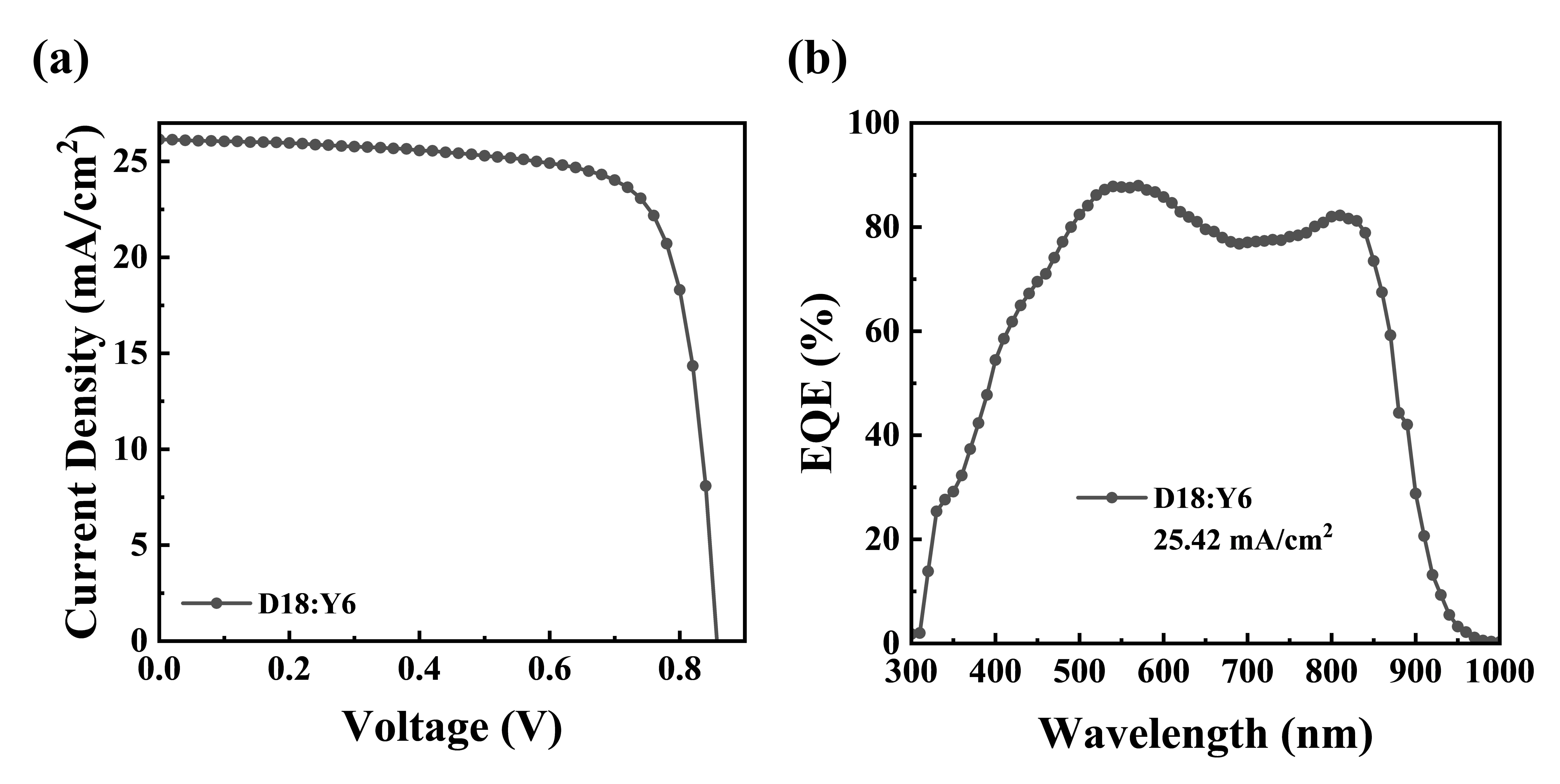


**Figure S13.** (a) *J-V* and (b) EQE curve of the single-junction organic solar cell.

**Table S1.** Optical properties of 2TC-F and TTC-F.

| Polymer HTM | $\text{λ}_{\text{max}}^{\text{film}}$ (nm) | | $\text{λ}_{\text{onset}}^{\text{film}}$ (nm) | $\text{E}_{\text{g}}^{\text{opt}}$ (eV) | $\text{E}_{\text{HOMO}}$ (eV) | $\text{E}_{\text{LUMO}}$ (eV) |
| --- | --- | --- | --- | --- | --- | --- |
| 2TC-F | 540 | | 637 | 1.95 | -5.56 | -3.61 |
| TTC-F | 559 | 665 | | 1.86 | -5.64 | -3.78 |

**Table S2.** Detailed parameters of the in-plane (100) direction from GIWAXS.

| HTM | (100) (Å) | FWHM (Å) | d-spacing (Å) | CCL (Å) |
| --- | --- | --- | --- | --- |
| 2TC-F | 0.287 | 0.0432 | 21.89 | 135.26 |
| TTC-F | 0.291 | 0.0395 | 21.59 | 147.93 |

**Table S3.** Detailed parameters of the out-of-plane (010) direction from GIWAXS.

| HTM | (010) (Å) | FWHM (Å) | d-spacing (Å) | CCL (Å) |
| --- | --- | --- | --- | --- |
| 2TC-F | 1.668 | 0.1556 | 3.77 | 37.55 |
| TTC-F | 1.640 | 0.1561 | 3.83 | 37.43 |

**Table S4**. Kinetic fitting parameters of CsPbI_2_Br, CsPbI_2_Br/2TC-F, and CsPbI_2_Br/TTC-F.

| Sample | τ_1_ (ns) | A_1_ (%) | τ_2_ (ns) | A_2_ (%) | τ_avg_ (ns) |
| --- | --- | --- | --- | --- | --- |
| CsPbI_2_Br | 0.13 | 72.63 | 1.75 | 27.37 | 1.49 |
| CsPbI_2_Br/2TC-F | 0.09 | 69.08 | 1.63 | 30.92 | 1.46 |
| CsPbI_2_Br/TTC-F | 0.07 | 40.07 | 1.31 | 59.93 | 1.27 |

**Table S5.** Photovoltaic parameters of CsPbI_2_Br PSCs based on solutions with different concentrations of 2TC-F and TTC-F.

| HTM | Concentration (mg mL^-1^) | *V*_OC_ (V) | *J*_SC_ (mA cm^-2^) | FF (%) | PCE (%) |
| --- | --- | --- | --- | --- | --- |
| 2TC-F | 6 | 1.367 | 14.20 | 72.97 | 14.17 |
|  | 8 | 1.386 | 14.12 | 74.30 | 14.54 |
|  | 10 | 1.363 | 13.77 | 65.53 | 12.22 |
| TTC-F | 8 | 1.390 | 15.06 | 81.06 | 16.97 |
|  | 10 | 1.415 | 15.01 | 82.77 | 17.58 |
|  | 12 | 1.383 | 14.06 | 81.60 | 15.87 |

**Table S6.** Photovoltaic parameters of CsPbI_2_Br PSCs with PTAA or Spiro-OMeTAD as HTM.

| HTM | *V*_OC_ (V) | *J*_SC_ (mA cm^-2^) | FF (%) | PCE (%) |
| --- | --- | --- | --- | --- |
| PTAA | 1.371 | 15.24 | 75.35 | 15.74 |
| Spiro-OMeTAD | 1.375 | 15.08 | 68.17 | 14.14 |

**Table S7.** Summary of fill factor loss calculation results.

| HTM | FF^DB^ (%) | FF_0_ (%) | FF (%) | ΔFF_1_ (%) | ΔFF_2_ (%) |
| --- | --- | --- | --- | --- | --- |
| 2TC-F | 91.85 | 86.78 | 74.18 | 5.07 | 12.48 |
| TTC-F | 91.85 | 88.08 | 82.77 | 3.77 | 5.31 |

**Table S8.** Photovoltaic parameters of single-junction organic solar cell.

| Active layer | *V*_OC_ (V) | *J*_SC_ (mA cm^-2^) | FF (%) | PCE (%) |
| --- | --- | --- | --- | --- |
| D18:Y6 | 0.857 | 26.15 | 76.20 | 17.08 |

**Table S9.** Photovoltaic parameters of the perovskite/organic tandem solar cells.

| HTM | *V*_OC_ (V) | *J*_SC_ (mA cm^-2^) | FF (%) | PCE (%) |
| --- | --- | --- | --- | --- |
| 2TC-F | 2.195 | 13.10 | 63.20 | 18.17 |
| TTC-F | 2.207 | 13.66 | 77.27 | 23.29 |

**References**

[1]. X. Li, A. Tang, Q. Guo, X. Guo, J. Chen, Q. Guo, M. Ji, Y. Meng, X. Li, and E. Zhou, *ACS Appl. Mater. Interfaces* **2022**, 14, 32308.
